# TCA cycle rewiring fosters metabolic adaptation to oxygen restriction in skeletal muscle from rodents and humans

**DOI:** 10.1038/s41598-017-10097-4

**Published:** 2017-08-29

**Authors:** Daniele Capitanio, Chiara Fania, Enrica Torretta, Agnese Viganò, Manuela Moriggi, Valentina Bravatà, Anna Caretti, Denny Z. H. Levett, Michael P. W. Grocott, Michele Samaja, Paolo Cerretelli, Cecilia Gelfi

**Affiliations:** 1 Department of Biomedical Sciences for Health, University of Milan, Segrate, (MI) Italy; 2 UO Proteomica Clinica, IRCCS Policlinico San Donato, San Donato Milanese, (MI) Italy; 3 CNR-Institute of Bioimaging and Molecular Physiology, Cefalù, (PA) and Segrate, (MI), Italy; 4 Department of Health Sciences, University of Milan, Milan, Italy; 5 Centre for Altitude, Space, and Extreme Environment Medicine, University College London (UCL), Institute of Child Health, University College London, London, UK; 6 Anaesthesia and Critical Care Research Unit, University Hospital Southampton, NHS Foundation Trust, Southampton, UK; 7 Integrative Physiology and Critical Illness Group, Division of Clinical and Experimental Science, Faculty of Medicine, University of Southampton, Southampton, UK

## Abstract

In mammals, hypoxic stress management is under the control of the Hypoxia Inducible Factors, whose activity depends on the stabilization of their labile α subunit. In particular, the skeletal muscle appears to be able to react to changes in substrates and O_2_ delivery by tuning its metabolism. The present study provides a comprehensive overview of skeletal muscle metabolic adaptation to hypoxia in mice and in human subjects exposed for 7/9 and 19 days to high altitude levels. The investigation was carried out combining proteomics, qRT-PCR mRNA transcripts analysis, and enzyme activities assessment in rodents, and protein detection by antigen antibody reactions in humans and rodents. Results indicate that the skeletal muscle react to a decreased O_2_ delivery by rewiring the TCA cycle. The first TCA rewiring occurs in mice in 2-day hypoxia and is mediated by cytosolic malate whereas in 10-day hypoxia the rewiring is mediated by Idh1 and Fasn, supported by glutamine and HIF-2α increments. The combination of these specific anaplerotic steps can support energy demand despite HIFs degradation. These results were confirmed in human subjects, demonstrating that the TCA double rewiring represents an essential factor for the maintenance of muscle homeostasis during adaptation to hypoxia.

## Introduction

Despite a number of studies on cell models and on human subjects exposed to short and long term hypoxia the biochemical mechanisms adopted by muscle tissue to cope with oxygen restriction are not fully understood. Overall, in mammals, hypoxic stress management is under the control of a class of transcriptional activators, the Hypoxia Inducible Factors (HIFs), whose activity depends on the stabilization of their labile α subunit^1^. In normoxia, the α subunits of HIFs undergo destabilization through hydroxylation by a family of dioxygenases named prolyl hydroxylase domain-containing enzymes (Phds)^2^. Phd1, 2 and 3 regulate HIFs intracellular levels and specifically, Phd2 act as master regulator of hypoxia response^3, 4^. HIFs degradation through Phds activity depends primarily on O_2_ levels, and on α-ketoglutarate (α-KG) and Fe^2+^ as molecular cofactors, underlining the interdependence between O_2_ delivery and cellular metabolism^2, 5–7^. However, HIFs stabilization, regulated by O_2_ levels, varies according to tissues’ tolerance of hypoxia. In particular, the skeletal muscle reacts to changes in substrates and O_2_ delivery by tuning its metabolism modulating HIFs. A characteristic of the muscle tissue is its plasticity, defined as the capacity to modulate metabolic pathways in response to changed functional demand under physiological or pathological conditions (i.e. under physical exercise or immobilization)^8–13^.

Previous proteomic studies conducted on human skeletal muscles exposed to various degrees of hypoxia, indicate that short and long term hypoxia exposures do not permanently activate HIF^14, 15^. Furthermore, the study of human vastus lateralis muscle exposed to severe hypoxia for 19 and 60 days in the course of the Caudwell Xtreme Everest expedition^16^ suggested a new mechanism of adaptation to hypoxia, indicating a metabolic TCA cycle rewiring and the use of glutamine as an alternative source for energy production^15^. Unfortunately, due to the paucity of muscle tissue, enzyme activities and detailed features could not be determined, leaving some aspects of muscle adaptation and particularly the molecular changes associated with the duration of hypoxia exposure, unexplained. To clarify the role of muscle metabolism in hypoxia, animal models represent a valuable source of information that can be translated to human samples^17–19^ possibly contributing not only to our understanding of the physiological mechanism of human hypoxia adaptation but also to the molecular mechanisms of hypoxia-associated diseases, including cardiovascular and pulmonary disorders and critical illness.

Unexpectedly the activation of HIFs varies in different animal models: HIF is not activated in hearts of mice in acute or chronic exposure^20^ whereas, rats adapt to acute and chronic oxygen shortage by activating HIF program and increasing anaerobic metabolism^21^ suggesting that HIFs stabilization, under comparable oxygen exposures, is species dependent^22^.

The aim of the present study is to provide a comprehensive overview of metabolic adaptation to hypoxia utilizing a mouse model that could reflect the molecular mechanism of hypoxia adaptation of the human skeletal muscle. The investigation was carried out combining proteomic analysis for the primary identification of key factors regulating the complexity of muscle changes under 2- and 10-day hypoxia and mRNA transcripts analysis, by qRT-PCR, followed by protein detection by antigen antibody reactions. Results from the mouse model were verified on protein extracts from human healthy subjects enrolled in previous independent studies^14–16^ and exposed to hypoxic conditions for 7/9 days at the Margherita Hut, Mt. Rosa (4,559 m a.s.l.), and for 19 days at the Mt. Everest base camp (5,300 m a.s.l.), respectively. The results demonstrate that both mice and human subjects exposed to hypoxia utilized the TCA cycle rewiring to cope with oxygen deficiency introducing for the first time in mammals an alternative use of the TCA cycle to specifically sustain energy production under conditions of limited oxygen availability. These results highlight the capacity of the human body to utilize unconventional metabolic pathways to sustain energy demand and maintain tissue homeostasis.

## Results

### HIF-1α and HIF-2α signalling

**Figure 1:**
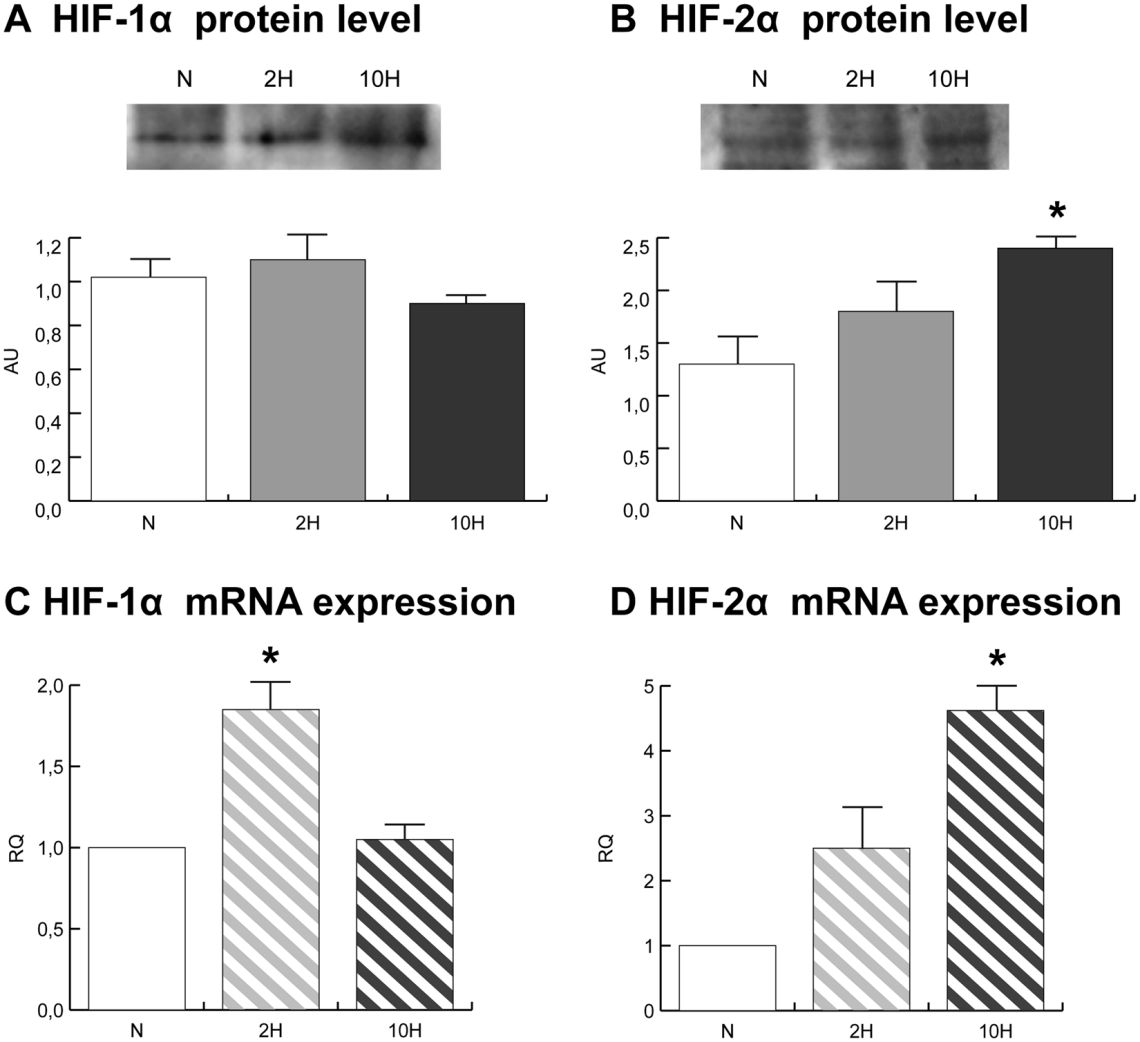
HIF-1α (**A**) and HIF-2α (**B**) immunoblotting and quantitation (cropped images; full lenght blots are included as Supplementary Data). qRT-PCR of HIF-1α (**C**) and HIF-2α (**D**) mRNA. Data were generated from eight independent experiments and values were referred to mRNA levels of control normoxic mice and expressed as mean ± SD (N, normoxic control; 2 H, 2-day hypoxia; 10 H, 10-day hypoxia). Immunoblotting and qRT-PCR data were subjected to ANOVA and Tukey test (n = 5, p < 0.05). Differences between 2 H and 10 H *versus* N were indicated with the * symbol.

HIF-1α was quantified in total extracts from gastrocnemius muscle of normoxic (N), 2-day hypoxic (2 H) and 10-day hypoxic (10 H) mice. No changes in HIF-1α protein levels were detected in skeletal muscle (Fig. 1A). HIF-1α mRNA expression increased in 2 H only (Fig. 1C).

HIF-2α protein and mRNA were significantly increased in 10 H (Fig. 1B and D) whereas a similar pattern of change, but of lesser magnitude, did not reach statistical significance in 2 H.

### Energy sensing and energetic metabolism regulators

Energy sensing was assessed by monitoring the level of phosphorylated (active) form of AMP-activated protein kinase (pAMPK) in relation to the total (AMPK). Metabolic reprogramming in response to glucose and fatty acid availability was assessed by monitoring the level of peroxisome proliferator-activated receptor-gamma coactivator 1α (PGC1α).

**Figure 2:**
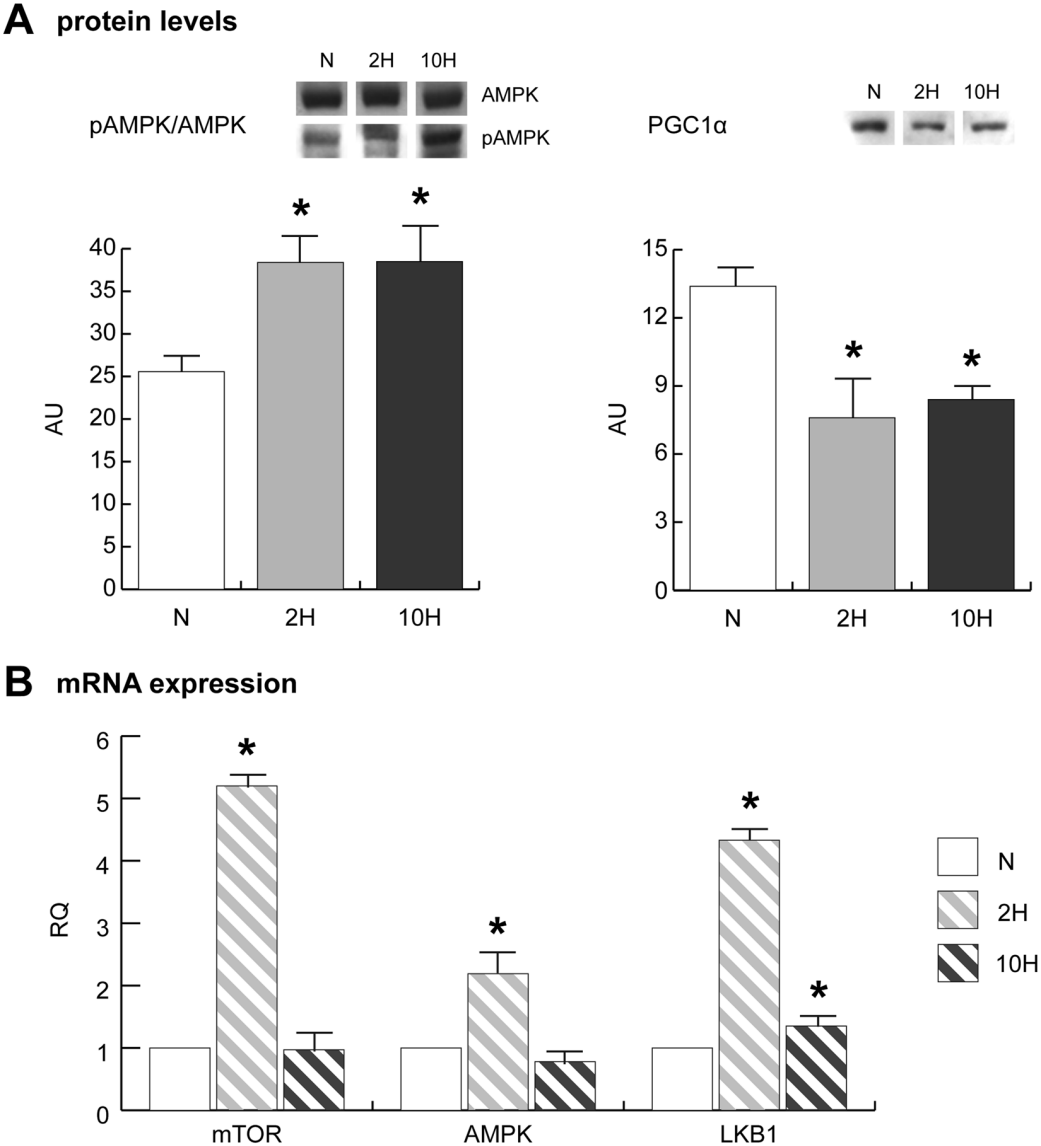
(**A**) pAMPK/AMPK and PGC1α immunoblotting and quantitation (cropped images; full lenght blots are included as Supplementary Data). (**B**) qRT-PCR of mTOR, AMPK and LKB1 mRNAs. Data were generated from eight independent experiments and values were referred to mRNA levels of control normoxic mice and expressed as mean ± SD (N, normoxic control; 2 H, 2-day hypoxia; 10 H, 10-day hypoxia). Immunoblotting and qRT-PCR data were subjected to ANOVA and Tukey test (n = 5, p < 0.05). Differences between 2 H and 10 H *versus* N were indicated with the * symbol.

The pAMPK/AMPK ratio increased, whereas PGC1α decreased, in both 2 H and 10 H skeletal muscles (Fig. 2, panel A).

The mRNA expression of serine/threonine-protein kinase mTOR and AMPK significantly increased specifically in 2 H skeletal muscle, whereas serine/threonine-protein kinase STK11 (LKB1) significantly increased in both 2 H and 10 H skeletal muscles; the increase in 2 H skeletal muscle was significantly higher than that observed in 10 H (Fig. 2, panel B).

### Skeletal muscle proteome profile

The proteome profiles of skeletal muscles from N, 2 H and 10 H were obtained through 2D-DIGE analysis and ESI MS/MS mass spectrometry.

Fifty-one spots were significantly changed comparing 2 H versus N skeletal muscle; conversely 70 spots were significantly changed comparing 10 H *vs*. N and 38 comparing 2 H *vs*. 10 H skeletal muscles. Overall, 42% of the identified spots were metabolic enzymes, while the remaining 58% included structural, myofibrillar, stress response and other proteins pertaining to a variety of cellular processes.

The identified metabolic proteins are illustrated in the representative proteomics maps in the Supplementary Fig. S1 and listed in Supplementary Table S1 with the corresponding UniProtKB accession numbers, cellular function, degree of variation expressed as average ratio and detailed mass spectrometry data.

Proteomic results indicate that protein dysregulation took place in three major cell compartments: metabolism, antioxidant proteins and cytoskeletal re-organization.

### Cell metabolism

**Figure 3:**
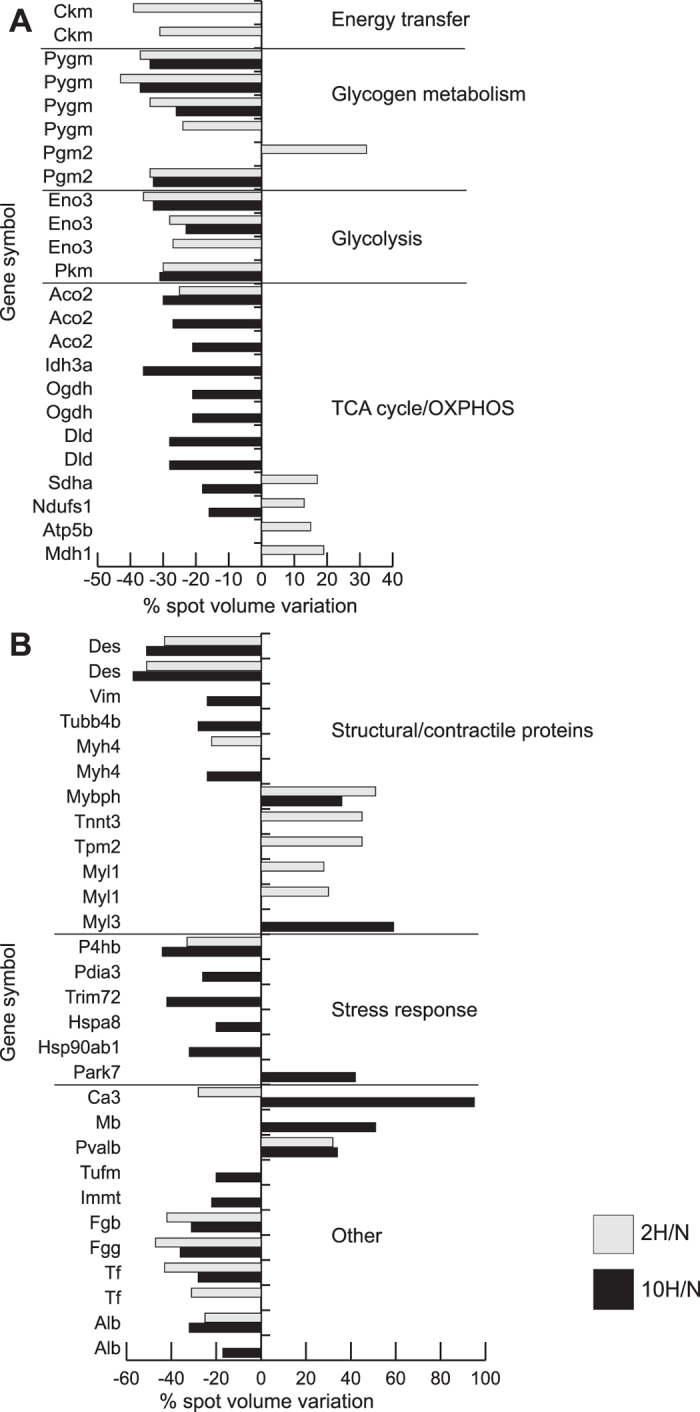
Proteomic profile of mice skeletal muscle exposed to 2-day (2 H) and 10-day (10 H) hypoxia. Histograms of skeletal muscle metabolic changes in 2 H (grey bars) and 10 H (black bars) as detected by 2D-DIGE analysis (ANOVA coupled to Tukey’s multiple-group comparison test, n = 5, p < 0.01). Protein spots statistically altered are reported with the corresponding gene name and the degree of variation is expressed as a percent of spot volume variation in hypoxic mice versus controls. (**A**) Metabolic proteins grouped according to their function: energy transfer enzymes (Ckm, creatine kinase),glycogen metabolism enzymes (Pygm, glycogen phosphorylase; Pgm2, phosphoglucomutase), glycolytic enzymes (Eno3, enolase; Pkm, pyruvate kinase), TCA cycle/OXPHOS enzymes (Aco2, aconitate hydratase; Idh3a, isocitrate dehydrogenase 3; Ogdh, 2-oxoglutarate dehydrogenase; Dld dihydrolipoyl dehydrogenase; Sdha, succinate dehydrogenase flavoprotein; Ndufs1, NADH dehydrogenase (ubiquinone) Fe-S protein 1; Atp5b, ATP synthase subunit beta; Mdh1, cytoplasmic malate dehydrogenase). (**B**) Structural/contractile (Des, desmin; Vim, vimentin; Tubb4b, tubulin beta-2C chain; Myh4, myosin-4; Mybph, myosin-binding protein H; Tnnt3, troponin T fast; Tpm2, tropomyosin beta chain; Myl1, myosin light chain 1/3, skeletal muscle isoform (MLC 1F); Myl3, myosin light chain 3 (MLC 1 sb)), stress response (P4hb, protein disulfide-isomerase; Pdia3, protein disulfide-isomerase A3; Trim72, tripartite motif-containing protein 72; Hspa8, heat shock cognate 71 kDa protein; Hsp90ab1, heat shock protein 84b; Park7, protein DJ-1) and other proteins (Ca3, Carbonic anhydrase 3; Mb, myoglobin; Pvalb, parvalbumin alpha; Tufm, elongation factor Tu, mitochondrial; Immt, Immt protein; Fgb, fibrinogen beta chain; Fgg, fibrinogen gamma chain; Tf, serotransferrin; Alb, serum albumin).

The expression levels of metabolic enzymes involved in energy transfer, glycogen metabolism, glycolysis and tricarboxylic acid (TCA) cycle were altered in both 2 H and 10 H (Fig. 3A).

Decrement of two proteoforms of creatine kinase M-type (Ckm) (−39%, −31%) characterized 2 H only, whereas decrement in glycogen utilization and glycolysis was a common feature of 2 H and 10 H. Specifically, glycogen phosphorylase (Pygm) decreased in 2 H (four proteoforms: −37%, −43%, −34%, −24%) and in 10 H (three proteoforms: −34%, −37%, −26%); phosphoglucomutase 2 (Pgm2) changed in 2 H (one proteoform increased, +32%, another one decreased, −34%) and in 10 H (−33%). In addition, several proteoforms of β-enolase (Eno3) decreased in both 2 H (−36%, −28%, −27%) and 10 H (−33%, −23%) as well as pyruvate kinase (Pkm) that was decreased by 30% under both conditions.

With regards to aerobic metabolism, NADH-ubiquinone oxidoreductase 75 KDa subunit (Ndufs1, 13%), succinate dehydrogenase flavoprotein (Sdha, 17%), ATP synthase subunit beta (Atp5b, 15%), and the cytoplasmic malate dehydrogenase (Mdh1, 19%) were increased in 2 H, whereas aconitate hydratase was decreased (Aco2, −25%). By contrast, in 10 H, Ndufs1 (−16%), Sdha (−18%), three proteoforms of Aco2 (−30%, −27%, −31%), the subunit alpha of mitochondrial isocitrate dehydrogenase 3 (Idh3, −36%), two proteoforms of 2-oxoglutarate dehydrogenase (Ogdh, both proteoforms −21%) and two proteoforms of dihydrolipoyl dehydrogenase (Dld, both proteoforms −28%) decreased.

### Structural/contractile and antioxidant defense molecules: proteomic results

Cytoskeletal remodelling (Fig. 3B) was suggested by a decrease of tubulin beta-2C chain (Tubb4) and vimentin (Vim) in 10 H, whereas desmin (Des) was decreased in both 2 H and 10 H.

Concerning contractile proteins: myosin-4 (Myh4) decreased in both conditions. Tubb4b, troponin T fast (Tnnt3), tropomyosin beta chain (Tpm2), myosin light chain 1/3, skeletal muscle isoform (Myl1) increased in 2 H, whereas myosin-binding protein H (Mybph) and myosin light chain 3 (Myl3) increased in 10 H.

Importantly, protein disulfide-isomerase (P4hb) decreased both in 2 H and 10 H. Protein disulfide-isomerase A3 (Pdia3), tripartite motif-containing protein 72 (Trim72), stress response protein HSP 90-beta (Hsp90ab1) and heat shock cognate 71 kDa protein (Hspa8) decreased in 10 H, whereas protein DJ-1 (Park7) increased.

### Other proteins

Carbonic anhydrase 3 (Ca3) increased in 10 H and decreased in 2 H, whereas myoglobin (Mb) increased in 10 H and parvalbumin alpha (Pvalb) increased in 2 H and 10 H. The mitochondrial elongation factor Tu (Tufm) and Immt protein (Immt) decreased in 10 H, whereas fibrinogen beta chain (Fgb), fibrinogen gamma chain (Fgg), serotransferrin (Tf), and serum albumin (Alb) decreased in both conditions (Fig. 3B).

### Citrate utilization

Citrate can be transported from mitochondria to cytoplasm to produce acetyl-CoA for fatty acid synthesis and oxaloacetate or, alternatively, it can be a substrate for α-ketoglutarate (α-KG) production through the cytosolic isocitrate dehydrogenase 1.

α-KG is a key metabolite being both a substrate for HIF prolyl hydroxylases and a precursor for glutamate production, which in turn can be utilized for glutamine and glutathione synthesis.

**Figure 4:**
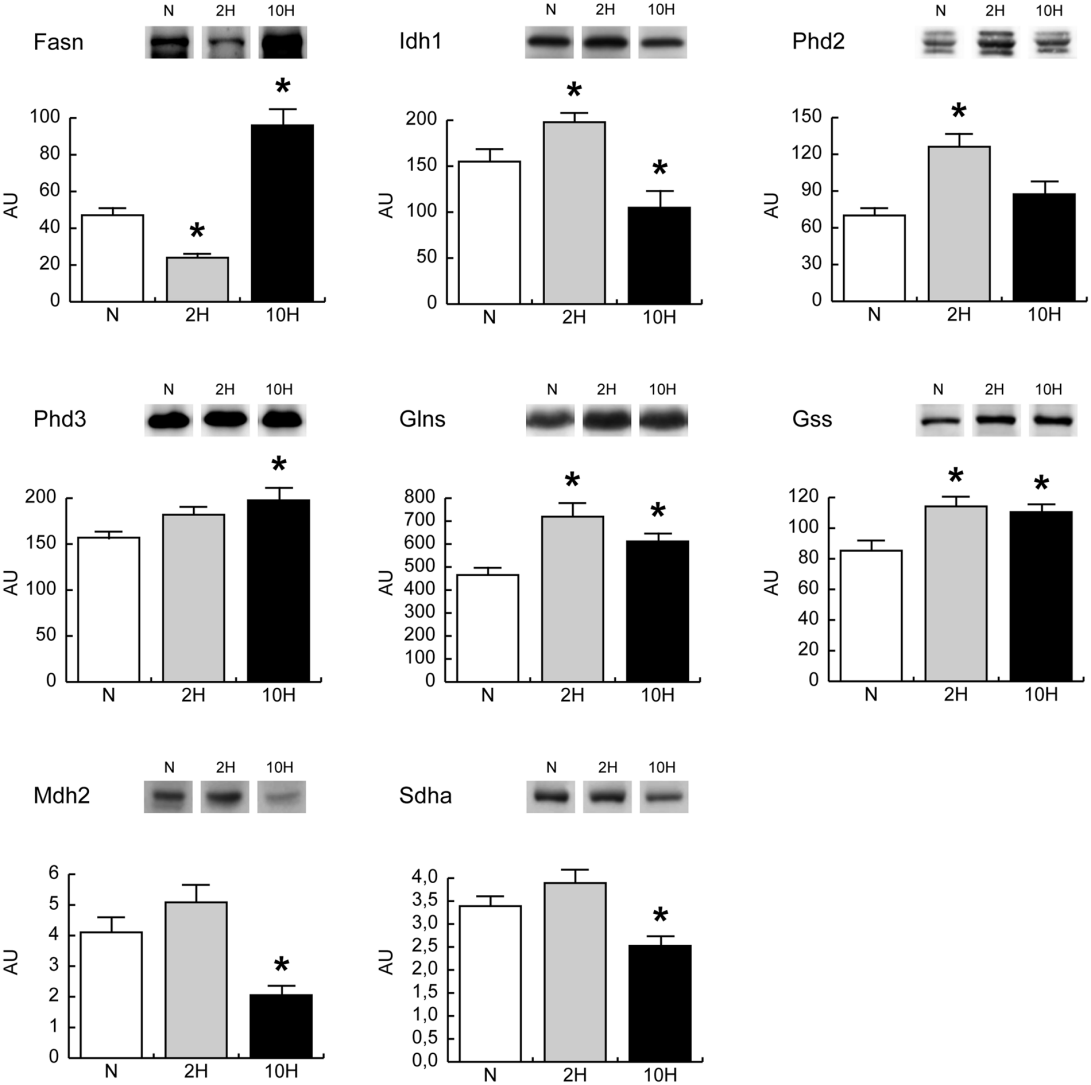
Expression levels of key metabolic enzymes in skeletal muscle of mice exposed to 2-day (2 H) and 10-day (10 H) hypoxia. Representative immunoblot images (cropped images; full lenght blots are included as Supplementary Data) and histograms of protein expression levels of fatty acid synthase (Fasn), isocitrate dehydrogenase 1 (Idh1), glutamine synthetase (Glns), glutathione synthetase (Gss), HIF prolyl hydroxylase 2 and 3 (Phd2 and Phd3), mitochondrial malate dehydrogenase (Mdh2) and succinate dehydrogenase A chain (Sdha) in 2 H and 10 H compared to normoxic controls (N). Data were normalized against the total amount of loaded proteins stained with Sypro Ruby. Statistical analysis was performed by ANOVA and Tukey test (n = 5, p < 0.05). *Significant difference between 2 H and 10 H *versus* N.

The expression levels of fatty acid synthase (Fasn), isocitrate dehydrogenase 1 (Idh1), HIF prolyl hydroxylases 2 and 3 (Phd2 and Phd3), l-glutathione synthetase and glutamine synthetase (Glns and Gss) were evaluated by immunoblotting, in skeletal muscle (Fig. 4). In 2 H, Fasn was decreased whereas an increment was observed for Idh1, Glns, Gss and Phd2. Conversely, in 10 H, the expression level of Fasn was markedly increased, while Idh1 was decreased. Glns and Gss remained increased compared to normoxic controls, Phd3 increased whereas Phd2 appeared normalized.

Beside fatty acid synthesis, citrate can generate cytosolic oxaloacetate. In a non-gluconeogenetic tissue such as muscle, oxaloacetate can be used through anaplerotic malate production by the cytosolic malate dehydrogenase (Mdh1). Cytosolic malate can be reimported into mitochondria and interconverted by mitochondrial malate dehydrogenase (Mdh2), fumarase and succinate dehydrogenase, refueling the TCA cycle. Proteomic data indicated a slight increment of Mdh1, succinate dehydrogenase A chain (Sdha), NADH ubiquinone oxidoreductase 75 KDa (Ndufs1) and ATP synthase beta (Atp5b) in 2 H and a decrement of Ndufs1 and Sdha in 10 H, in spite of a decrease of mitochondrial isocitrate dehydrogenase 3 (Idh3) and aconitate hydratase (Aco2) in both conditions (Fig. 3A). To demonstrate the use of this alternative pathway to produce energy, the expression levels of Mdh2 and Sdha were assessed by antigen antibody reactions (Fig. 4). Results showed a trend to increase in 2 H, and a statistically significant decrement in 10 H that confirmed proteomic data.

**Figure 5:**
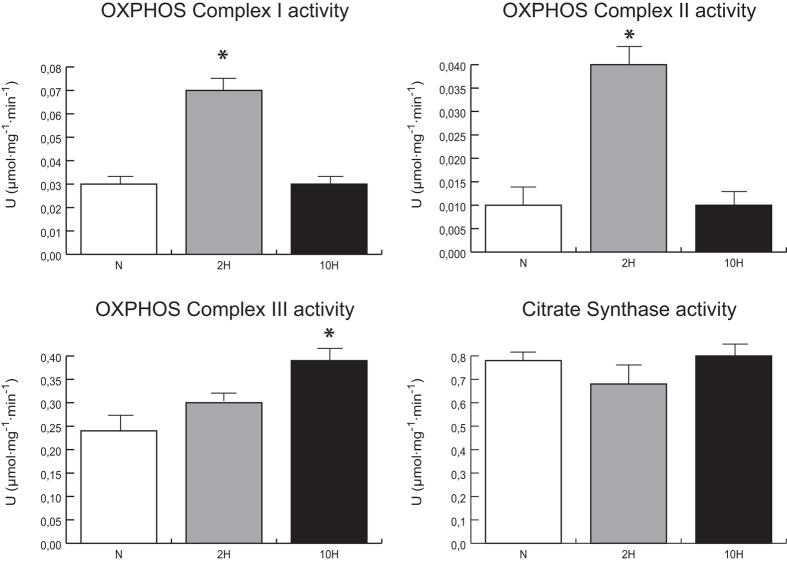
Enzymatic activity of respiratory chain complexes. Histograms showing NADH dehydrogenase (OXPHOS Complex I), succinate dehydrogenase (OXPHOS complex II), ubiquinol-cytochrome-c reductase (OXPHOS complex III) and citrate synthase (a marker of mitochondrial content) enzymatic activities in 2-day (2 H) and 10-day (10 H) hypoxia mice compared to normoxic controls (N). Activities were referred to the total protein content of the samples (Units = µmol/mg/min). Mean ± SD, ANOVA and Tukey test, n = 5, *p < 0.05.

Concerning the respiratory chain, complex I and II activities were increased in 2 H compared with N, whereas complex III remained unchanged. In 10 H, complex I and II were unchanged with respect to controls and complex III increased. Citrate synthase activity remained unchanged in both 2 H and 10 H vs. N (Fig. 5).

### Hexosamine pathway

Since the activation of glutamine synthetase and the restriction of α-KG flux through the TCA were observed, it can be postulated that glutamine is available either as substrate precursor or for the hexosamine pathway.

The hexosamine biosynthetic pathway (HBP) is considered a sensor for the nutritional state of the cell, as this metabolic pathway integrates glucose, glutamine, fatty acids and uridine. It provides UDP-N-acetylglucosamine (UDP-GlcNAc), the monosaccharide donor molecules for O-GlcNAcylation or N-glycosylation. The latter is necessary for ER homeostasis and contributes to protein tertiary structure formation and protein sorting to achieve proper protein folding in the ER. Disruption of this glycosylation leads to the accumulation of unfolded proteins in the ER lumen and induces the unfolded protein response (UPR)^23^. The HBP relies on glucose and glutamine uptake.

**Figure 6:**
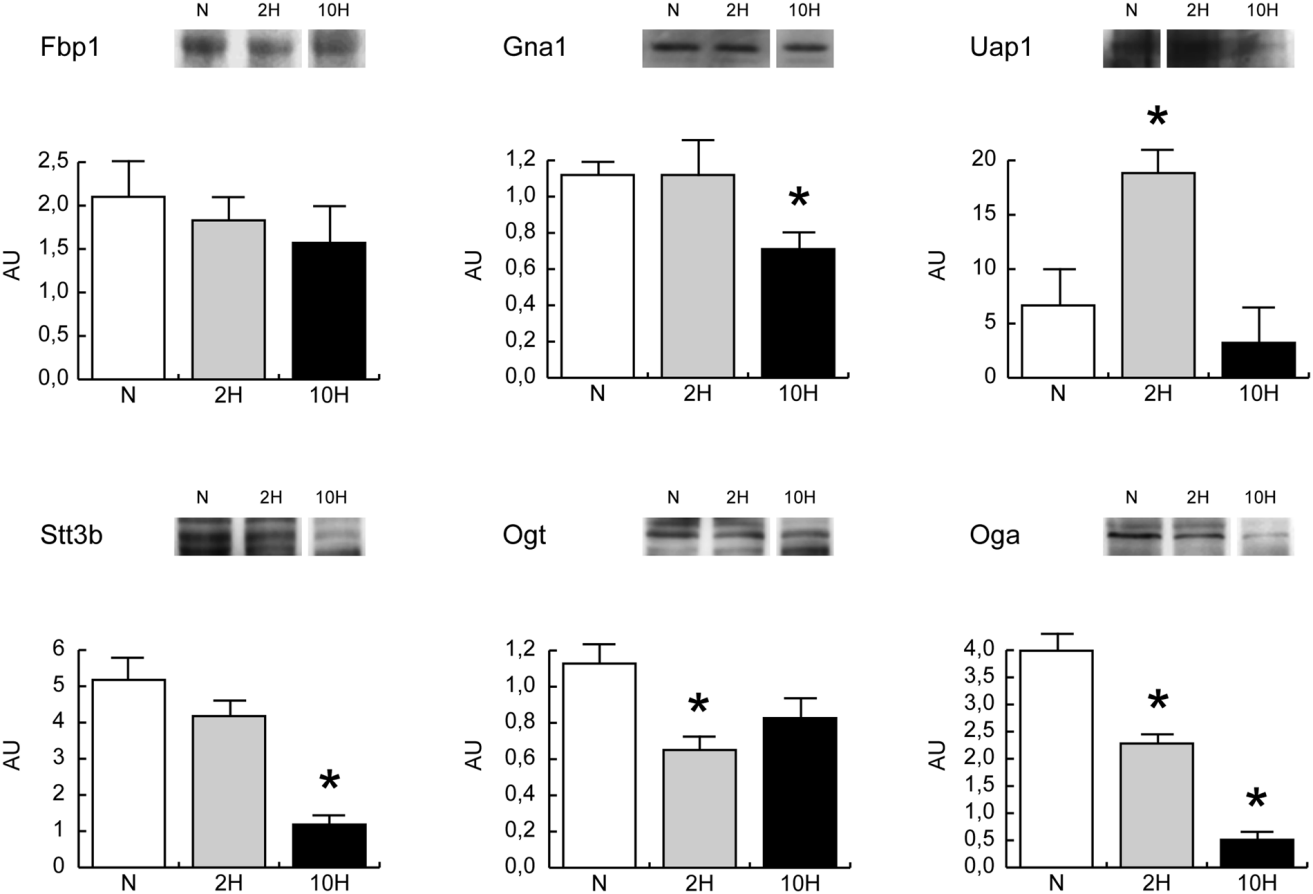
Expression levels of hexosamine pathway enzymes. Representative immunoblot images (cropped images; full lenght blots are included as Supplementary Data) and histograms of protein expression levels of fructose-1,6-bisphosphatase (Fbp1), glucosamine 6-phosphate N-acetyltransferase (Gna1), UDP-N-acetylhexosamine pyrophosphorylase (Uap1), Dolichyl-diphosphooligosaccharide-protein glycosyltransferase subunit STT3B (Stt3b), UDP-N-acetylglucosamine-peptide N-acetylglucosaminyltransferase 110 kDa subunit (Ogt) and protein O-GlcNAcase (Oga) in 2-day (2 H) and 10-day (10 H) hypoxia mice compared to normoxic controls (N). Data were normalized against the total amount of loaded proteins stained with Sypro Ruby. Statistical analysis was performed by ANOVA and Tukey test (n = 5, p < 0.05). *Significant difference between 2 H and 10 H *versus* N.

Fructose-1,6-bisphosphatase (Fbp1) was unchanged, both in 2 H and 10 H conditions. In 2 H, the HBP pathway analysis indicated glucosamine 6-phosphate N-acetyltransferase (Gna1) similar to normoxic control whereas UDP-N-acetylhexosamine pyrophosphorylase (Uap1) was increased. Dolichyl-diphosphooligosaccharide-protein glycosyltransferase subunit STT3B (Stt3b) was unchanged, whereas UDP-N-acetylglucosamine-peptide N-acetylglucosaminyltransferase 110 kDa subunit (Ogt) and protein O-GlcNAcase (Oga) were decreased. In 10 H, Gna1 decreased whereas Uap1 and Ogt were normalized. Stt3b and Oga were decreased (Fig. 6).

These results suggest that glutamine in 2 H can be utilised for UDP-N-acetylglucosamine synthesis whereas in 10 H it might become available for anabolic substrate production or glutathione synthesis.

### Autophagy

**Figure 7:**
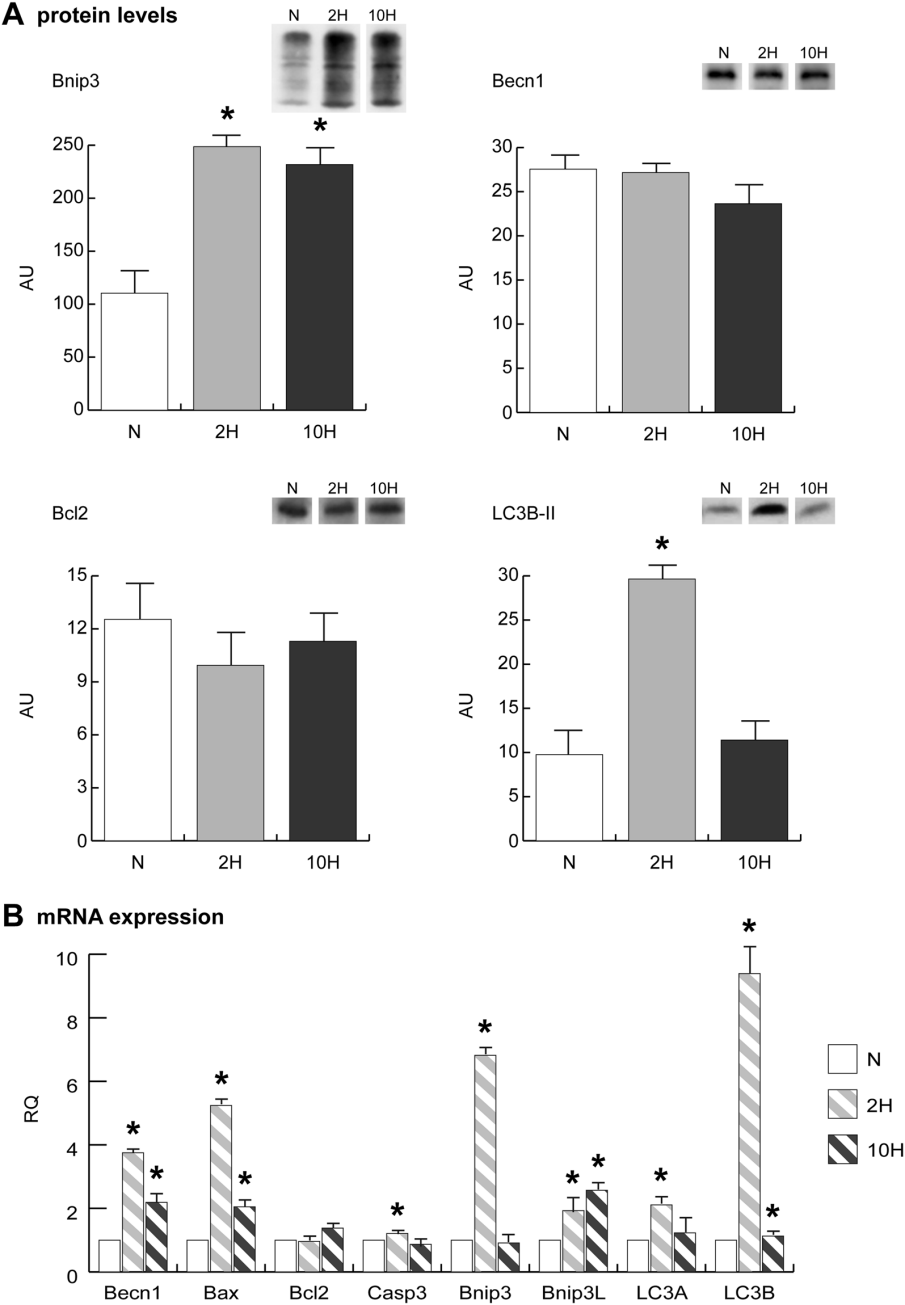
(**A**) BCL2/adenovirus E1B 19 kDa protein-interacting protein 3 (Bnip3), Beclin-1 (Becn1), Apoptosis regulator Bcl-2 (Bcl2) and the lipidated form of microtubule-associated protein 1 light chain 3 beta (LC3B-II) immunoblotting and quantitation (cropped images; full lenght blots are included as Supplementary Data). (**B**) qRT-PCR of Becn1, apoptosis regulator Bax, Bcl2, Caspase-3 (Casp3), Bnip3, BCL2/adenovirus E1B 19 kDa interacting protein 3-like (Bnip3L), microtubule-associated protein 1 light chain 3 alpha (LC3A) and LC3B. Data were generated from eight independent experiments and values were referred to mRNA levels of control normoxic mice and expressed as mean ± SD. Immunoblotting and qRT-PCR data were subjected to a ANOVA and Tukey test (n = 5, p < 0.05). Differences between 2 H and 10 H *versus* N were indicated with the * symbol.

Cytoskeletal remodelling is associated with the activation of the autophagic process to clear damaged proteins. Immunoblottings indicated that the energy costly process of autophagy was increased as suggested by the increase of BCL2/adenovirus E1B 19 kDa protein-interacting protein 3 (Bnip3) level both in 2 H and 10 H. The lipidated (activated) form of microtubule-associated protein 1 light chain 3 beta (LC3B-II) was increased in 2 H and normalized in 10 H. The levels of Beclin1 (Becn1) and apoptosis regulator Bcl2 were unchanged, both in 2 H and in 10 H (Fig. 7A). These results were confirmed also at transcript level, with the exception of Becn1 mRNA, which was upregulated in both conditions. qRT-PCR indicated that microtubule-associated protein 1 light chain 3 alpha (LC3A), LC3B and Bnip3 increased in 2 H whereas Becn1, apoptosis regulator Bax and BCL2/adenovirus E1B 19 kDa interacting protein 3-like (Bnip3L) increased both in 2 H and 10 H (Fig. 7B).

### Rewiring of the TCA cycle in human skeletal muscle exposed to 7 to 9 days and 19 days severe hypoxia

**Figure 8:**
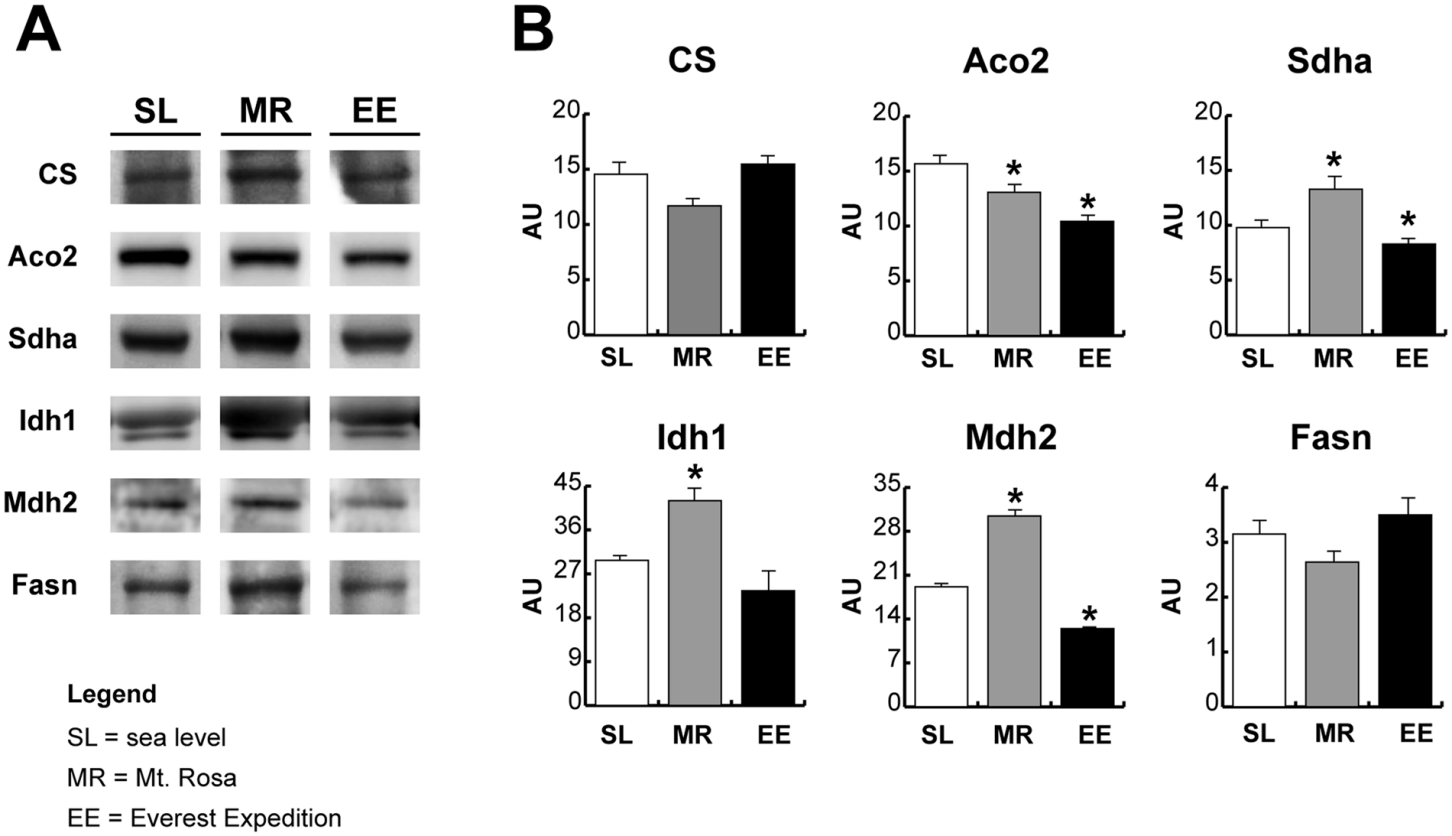
Expression levels of key metabolic enzymes in human *vastus lateralis* muscle after 7–9 days (Mt. Rosa, MR) and 19 days (Everest expedition, EE) of hypoxia exposure. Representative immunoblot images (cropped images; full lenght blots are included as Supplementary Data) and histograms of protein expression levels of citrate synthase (CS), aconitate hydratase 2 (Aco2), succinate dehydrogenase A chain (Sdha), isocitrate dehydrogenase 1 (Idh1), mitochondrial malate dehydrogenase (Mdh2) and fatty acid synthase (Fasn), in MR and EE compared to sea level (SL). Data were normalized against the total amount of loaded proteins stained with Sypro Ruby. Statistical analysis was performed by ANOVA and Tukey test (n = 6, p < 0.05). *Significant difference between MR and EE *versus* SL.

Markers of TCA rewiring, detected in 2 H and 10 H mice, were also determined in human skeletal muscle exposed to either 7 to 9 days hypoxia (Angelo Mosso laboratory, Margherita Hut, Mt. Rosa Italy, 4,559 m a.s.l.) or 19 days hypoxia (at the Everest base camp, Mt. Everest, 5,300 m a.s.l.) and are represented in Fig. 8. Results indicated non-significant changes in citrate synthase protein level after both exposures, a significant down regulation of Aco2 in both conditions, an increment of Sdha after 7 to 9 days exposure and a down regulation after 19 days at the Everest base camp. Idh1 and Mdh2 were increased after 7 to 9 days, whereas they were unchanged and decreased respectively, after 19 days at the Everest base camp. Fasn did not change significantly after both exposures. These results confirm the role of TCA cycle rewiring in adaptation to hypoxia and its dependency on the duration of hypoxia exposure.

## Discussion

This study addresses the role of TCA cycle during hypoxia adaptation of the skeletal muscle tissue in rodents and validates the results in human muscles.

In mice, the activity of Phds inhibits HIF-1α stabilization both in 2 H and 10 H, whereas HIF is active only in 10 H in which HIF-2α is increased, suggesting that metabolic adaptation is at variance with that observed in severely hypoxic cell cultures^1, 24–26^.

In muscle under physiological conditions, the response to energy demand is controlled by AMPK, whose activity increases during metabolic stress conditions induced by cellular ATP restriction. Upon activation, AMPK increases cellular energy levels by inhibiting anabolic energy consuming pathways (fatty acid synthesis, protein synthesis, etc.) and stimulating catabolic pathways (fatty acid oxidation, glucose transport, etc.). Furthermore, PGC1α stimulates mitochondrial biogenesis and promotes muscle tissue remodelling toward a more oxidative and less glycolytic fiber-type composition, and participates in the regulation of both carbohydrate and lipid metabolism^27^. Our results indicate that, despite AMPK signalling, in mice, PGC1α decreased in both 2 H and 10 H. These changes were observed also in the heart muscle exposed to the same hypoxia protocol in a previous study^20^. We believe that this profile is a transient state that precedes the activation of genes under HIFs control. This is possible due to the energy saving mechanisms since, by inhibiting PGC1α, the energy-costly process of the mitochondrial biogenesis is reduced. This phenomenon appears robust and characteristic of the early phase of adaptation to hypoxia, preceding HIFs activation and being associated to macroautophagy onset^28^. Autophagy can also explain the increment of respiratory chain complexes observed in the early stage. This event has been described also in hypoxic fibroblasts cultures^29^. In the presence of autophagy, the dysfunctional proteins are degraded, thus enzyme levels are reduced, despite their activities preserved. This can occur because the fusion and fission processes, at mitochondrial level, segregate and selectively discard damaged mitochondrial components, enriching the remaining mitochondria with undamaged material and thus contributing to the maintenance of the bioenergetics efficiency of the muscle cell^30, 31^. These results do not provide sufficient information on the energetic status of the tissue and on its adaptation to hypoxia. However, their integration with the proteomic analysis of primary metabolism, cytoskeletal remodelling and stress response, contributes to go better insight into both questions.

In the context of muscle energy demand, the tricarboxylic acid (TCA) cycle represents the metabolic link between carbohydrates, lipids, aminoacid oxidation and ATP production through oxidative phosphorylation. The metabolic profile of hypoxic skeletal muscle was characterized by lower expression levels of creatine kinase (Ckm) and of enzymes belonging to the glycogen metabolism (Pygm, Pgm2), glycolysis (Eno3, Pkm) and TCA cycle (Aco2, Idh3a, Ogdh, Dld), both in 2-day and in 10-day severe hypoxia. It is noteworthy that the drop of Ckm, involved in glycogen metabolism, and of the TCA cycle, is a common feature of human subjects exposed to short and long term extreme altitude, as described in previous proteomic differential studies of hypoxia adaptation^14, 15^. These studies indicated a decreased level of glycolytic enzymes in contrast with results on cultured cells or in rats under severe hypoxia^24, 32^.

The cause of hypoxic skeletal muscle harm has been mainly attributed to ROS production induced by oxidative stress^33, 34^. Several pieces of evidence have shown that proteins exposed to oxidants like hydrogen peroxide, superoxide, hydroxyl radical and peroxynitrite enhanced proteasomal degradation^35^. Creatine kinase, glycogen phosphorylase and pyruvate kinase as well as the ryanodine receptor 1^36^ were described as targets of oxidation of Cys residues and nitration^37^. In the present context, the role of mitochondrial ROS affecting calcium handling proteins involved in contractility, described in animal model^36^, can be associated to muscle mass loss observed in Caucasian exposed to high altitude^38, 39^.

Also, mitochondrial aconitate hydratase (Aco2) was under expressed both in 2- and 10-day severe hypoxia. This enzyme has been reported as being particularly susceptible to ROS-mediated inhibition^40, 41^ and, subsequently, to proteolytic degradation after oxidative modification. Aco2 decrement was also observed in previous studies of hypoxia adaptation in animal models^21^ and in the *vastus lateralis* muscle of humans exposed to hypobaric hypoxia^14^. It can be hypothesized that under-expression of the above-described molecules could be due to impaired translation or enhanced protein degradation to support the amino acid pool. Further investigations, addressing these aspects, are in progress.

Concerning Mdh2 and Sdha, the increased levels in 2 H and decreased levels in 10 H, assessed by immunoblotting, confirm proteomic data and suggest that the reverse TCA cycle utilizes citrate synthase to convert citrate into oxaloacetate and acetyl-CoA in 2 H, whereas in prolonged hypoxia lipid metabolism plays a prominent role.

To support TCA cycle rewiring, NADH is an essential cofactor and it can become available through a cytosolic accumulation mediated by enolases and pyruvate kinase decrement. The latter, by decreasing the availability of pyruvate, blunts its transformation to lactate, and thus the oxidation of NADH to NAD+, leaving the substrate available for cytoplasmic malate dehydrogenase. The resulting cytosolic malate can be reimported into mitochondria, thus refueling the TCA cycle.

Previous studies have indicated that during the transition from a short-term to prolonged hypoxia (i.e. Mt. Everest Pyramid, 5,400 m a.s.l.) human subjects, performing maximal exercise at high altitude, acutely exhibit reduced levels of blood lactate, that renormalize after a long stay at altitude. This unexpected transient phenomenon has been termed the “lactate paradox”^42–44^. Taking into consideration that mice have a five to seven-fold faster specific metabolic rate than humans, in our experimental conditions, lactate is not produced since enolases are decreased and NADH is utilized to support malate and succinate synthesis, and it is transient since the metabolic rewiring reflects the early phase of adaptation only. Further study, based on metabolomics analysis, could be useful to determine metabolites associated with the above-described TCA rewiring: these molecules, can be considered putative biomarkers for clinical monitoring of diseases associated with oxygen deprivation that occurs when patients experience intermittent hypoxia, such as in sleep apnoea syndrome^45^.

To sustain the metabolic demand, the first TCA rewiring occurs in mice in 2 H and is mediated by cytosolic malate whereas the second rewiring occurs in 10 H and is mediated by Idh1 and Fasn, supported by glutamine and HIF-2α increments. It is known that, under hypoxic stress, mitochondrial lipid oxidation is reduced in favor of lipid storage^46, 47^ and this process is modulated by the action of HIF-2α^48^. In humans, the first rewiring is observed after 7/9 days of hypoxia exposure whereas the second rewiring is revealed after 19 days of oxygen restriction. Our results also indicate a role for glutamine in the 10 H phase of adaptation. Glutamine, exerts many important metabolic functions^49^ like anaplerosis^50^ or *de novo* lipogenesis via a reductive metabolism^51, 52^. Since skeletal muscle is the main site of glutamine production as well as the major contributor to whole-body glutamine supply^53^, it is clear that effective glutamine synthesis contributes to ensure nutrient provision, anti-inflammatory and anti-oxidative responses, not only to muscular function^54, 55^. Our results indicate that glutamine could become available as an anabolic precursor of UDP-N-acetylglucosamine, as indicated by Uap1 upregulation in 2 H, or generate de novo lipogenesis utilizing the anaplerotic cycle trough Idh1 in 10 H.

This observation in 10 H is supported by the increment in fatty acid synthase (Fasn) that is a common outcome of prolonged hypoxia exposure in human skeletal muscle. Fasn uses products of the TCA cycle to synthesize the saturated fatty acid palmitate, contributing to *de novo* lipogenesis.

Overall, it can be concluded that in the early phase of adaptation muscle tissue utilizes recycled malate to refuel the TCA in mice and this is confirmed in human subjects exposed to 7/9 days hypoxia, whereas after 10 days in mice and 19 days in humans, a central role of glutamine can be postulated.

Concerning prolyl hydroxylase domain containing enzymes, it is known that both enzymes contribute to the HIF system regulation with different substrate specificity, Phd2 being more active towards HIF-1α and Phd3 towards HIF-2α^3, 56^. Here we confirmed the induction of Phd2 and Phd3 in skeletal muscle under 2-day hypoxia only. Their inducible behavior presumably reflects the tissue flexibility in regulating HIF-dependent response to hypoxia and this response is modulated in a time-dependent manner.

Another element, clearly disclosed by the present study, is the cytoskeletal re-arrangement that can be associated to autophagosome formation enhancing the protein quality control thus preserving tissue homeostasis and preventing ER stress^23^.

Our data suggest that the autophagic activation observed in 2 H is sustained by the metabolic rewiring that utilizes cytosolic malate dehydrogenase to refuel the TCA cycle thus maintaining tissue homeostasis. In contrast, the energetic status of 10 H cannot support autophagosome formation, being energy production impaired. These results provide a link among autophagic impairment, cytoskeletal remodelling and TCA cycle rewiring, to muscle homeostasis disruption^17–19^. For the first time this study identifies a modified TCA rewiring system in human and mouse muscles. These results could contribute to improve the understanding of the metabolic adaptation occurring in pathological conditions, including sleep apnea, cardiovascular and pulmonary disorders and critical illness.

Nevertheless we are aware that the small sample size and the absence of an independent replication for more confident statistics, besides the inability of 2D-DIGE to identify species under 125 picomoles, represent a major limitation of this study. More efforts should be implemented to increase the number of samples, recruiting not only healthy subjects exposed to low oxygen levels but also patients characterized by comparable hypoxic conditions, like sleep apnea syndrome, cardiovascular and pulmonary insufficiency. The validation step is essential to support the proposed adaptive molecular mechanism, overcoming eventual statistical bias and the contribution of confounding effects.

### Overall Conclusions

From the present study, it can be hypothesized that citrate, generated by pyruvate and the reductive TCA cycle, driven by cytosolic Idh1, could be utilized to produce ATP for energetic support. This occurs through an alternative pathway that utilizes the cytosolic malic enzyme to produce malate. These results suggest that the combination of two specific anaplerotic steps can support energy demand despite HIFs degradation, activating sophisticated mechanisms, including ROS signaling and autophagy, specific for tissue preservation. Interestingly, this mechanism is active in 2 H not in 10 H, where adaptation occurs by increasing fatty acid oxidation as indicated by Fasn increment and Idh1 decrement.

Overall these results suggest a direct interplay between ROS production and the double TCA cycle rewiring in which ROS may act as signalling molecules in concert with the metabolic rewiring, supported by a fine quality control exerted by autophagy.

These aspects should be considered in the frame of adaptive cell hypoxia, a transient phenomenon in which compensatory mechanisms sustain metabolism and energy utilization, in a changing environment^34, 57^. Mitochondrial ROS formation can be considered a potential effector of such a sensing system^58^. Low Po_2_ and high OXPHOS complexes levels, make electrons more available for reduction reactions (*e.g*. the reduction of O_2_ to superoxide), enhancing ROS production and triggering cell signaling to keep the energy demand at lower levels.

**Figure 9:**
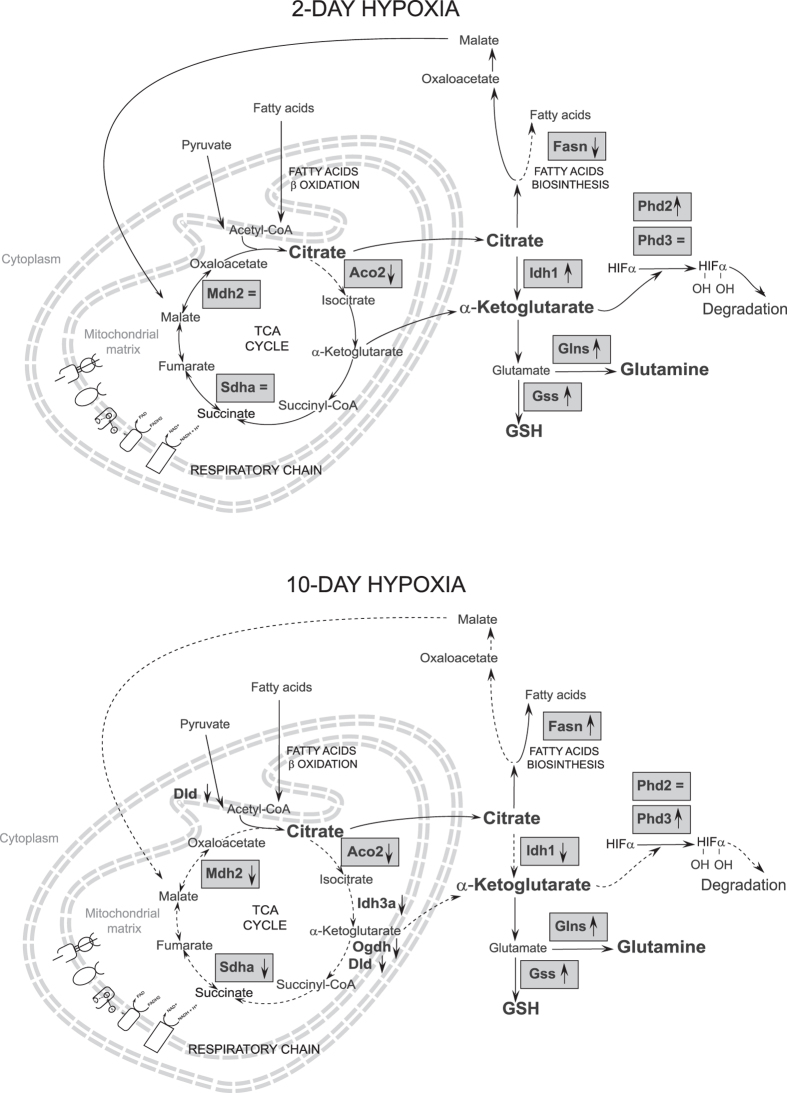
Schematic representation of the metabolic adaptation to 2-day hypoxia (2 H) and 10-day hypoxia (10 H) in skeletal muscle. Protein expression profiles derived from 2D-DIGE and immunoblotting analyses are reported in grey rectangles with the corresponding gene names and a symbol indicating their increase (↑), decrease (↓) or their similarity (=) in 2 H (upper panel) or in 10 H (lower panel) *versus* normoxic controls (Aco2, aconitate hydratase 2; Fasn, fatty acid synthase; Idh1, isocitrate dehydrogenase 1; Glns, glutamine synthetase; Gss, glutathione synthetase; Phd2 and Phd3, HIF prolyl hydroxylase 2 and 3, Mdh2, mitochondrial malate dehydrogenase; Sdha, succinate dehydrogenase A chain).

In conclusion, in 2-day hypoxia, cytosolic citrate is utilized for glutamine, glutathione and α-KG production to promote HIF prolyl hydroxylases activity and to sustain muscle adaptation to hypoxia. Conversely, after 10-day exposure cytosolic citrate is diverted to fatty acids biosynthesis (see Fig. 9). Importantly, these results are confirmed in human subjects exposed for 7/9 and 19 days, respectively, to severe hypoxia, demonstrating that the TCA double rewiring is also active in human subjects and represents an essential factor for the maintenance of muscle homeostasis during adaptation to hypoxia.

## Methods

### Animals

Mice were divided into three groups: controls, normoxic mice (N, n = 5), which breathed room air (FIO_2_ = 0.21), mice exposed for 48 h to normobaric hypoxic atmosphere (FIO_2_ = 0.08) (2 H, n = 5) and mice which were subjected to the same treatment as 2 H, but for 10 days (10 H, n = 5). The hypoxic chamber incorporated a compensation unit to prevent any exposure of animals to room air during feeding, cleaning operations and sacrifice^59^. For sacrifice, animals were transferred to the compensation chamber, which was continuously flushed with the hypoxic gas, and anesthetized with a hip injection of sodium thiopental (10 mg/100 g body wt) and heparin (500 units). Skeletal muscles were removed soon after the sacrifice (less than 1 min), immediately frozen in liquid nitrogen and then stored at −80 °C until use. The investigation was carried out in accordance with the Guide for the Care and Use of Laboratory Animals published by the US National Institutes of Health (NIH Publication No. 85-23, revised 1996). All experimental protocols were approved by the University of Milan ethical committee.

### Human biopsies

The participants were: (a) healthy volunteers recruited from the investigators group of the Caudwell Xtreme Everest 2007 research expedition to Mount Everest of the University College London (UCL), UK^15, 16^ or (b) members recruited by the Margherita Hut Scientific Committee at the University of Torino (UNITO), Italy^14^. Written informed consent was obtained from all participants. Protocols were approved by the UCL research ethics committee and the UNITO Ethical Committee in accordance to the World Medical Association Code of Ethics, Declaration of Helsinki (1964). Baseline skeletal muscle biopsies were taken from both groups prior to departure. Members of a) had a repeat biopsy taken on expedition day 19 (6 days after arrival at the Everest Base Camp, Mt. Everest, 5,300 m a.s.l.), whereas members of b) had a repeat biopsy taken over days 7–9 (3 days after arrival at the Angelo Mosso laboratory, Margherita Hut, Mt. Rosa, 4,559 m a.s.l.). Biopsies were taken from the mid part of the *vastus lateralis* muscle. A part of each biopsy (5–10 mg) was snap-frozen and maintained in liquid nitrogen until analysis in Milan.

### Protein extraction

Two-dimensional differences in gel electrophoresis (2D-DIGE) and immunoblots: an aliquot of each frozen muscle was suspended in lysis buffer (urea 7 M, thiourea 2 M, CHAPS 4%, Tris 30 mM, and PMSF 1 mM) and solubilized by sonication on ice. Proteins were selectively precipitated using PlusOne 2D-Clean-up kit to remove non-protein impurities and re-suspended in lysis buffer. Protein extracts were adjusted to pH 8.5 by the addition of NaOH 1 M solution, and sample concentrations were determined using PlusOne 2D-Quant kit.

Enzymatic activities: 30–50 mg of frozen muscle were diluted 1:20 (wt/vol) in ice-cold sucrose muscle homogenization buffer (Tris 20 mM, KCl 40 mM, EGTA 2 mM, Sucrose 250 mM, pH 7.4) and homogenized in a glass-glass tissue grinder. Muscle homogenates were centrifuged at 600 g for 10 min at 4 °C. Supernatants were immediately assayed for enzymatic activities. Total protein quantitation was determined using PlusOne 2D-Quant kit.

### Proteomic analysis

Protein labeling, 2D separation and analysis were performed as previously described^20^. The proteomic profile of each group of hypoxic mice was compared with the normoxic group. Statistically significant differences of 2D-DIGE data were computed by an independent one-way analysis of variance (ANOVA) coupled to Tukey’s multiple-group comparison test (p < 0.01). Normality of data distribution was assessed by applying the Shapiro-Wilk test. False discovery rate was applied as multiple test correction in order to keep the overall error rate as low as possible. Statistically changed spots were further filtered on the basis of the average ratio value provided by DeCyder 2D software: only spots with an average ratio >1.1 or <−1.1 were considered as differentially expressed.

Proteins were identified by matrix-assisted laser desorption/ionization time of flight utilizing the method previously described^20^.

Identified proteins are indicated in representative proteomics maps in Supplementary Fig. S1. Detailed proteomics and mass spectrometry data are reported in Supplementary Table S1.

### Real time PCR

Total RNA from frozen muscle was extracted by TRIzol Reagent according to the manufacturer’s protocol (Invitrogen) and residual phenol and salts were removed using the PureLink™ Micro-to Midi™ RNA Total RNA Purification System (Invitrogen). RNA quality and dosage were assessed on the Bioanalyzer instrument (Agilent Technologies): the A260/280 ratio and RNA Integrity Number (RIN) were measured. Samples in which RIN value was below 7.0 were unused for this study. About 1 µg of RNA sample was reverse transcribed to cDNA with SuperScriptII reverse transcriptase and oligo(dT) according to manufacturer’s specifications (Invitrogen). Primers for qRT-PCR analysis were designed by the Primer3 software (http:// fokker.wi.mit.edu/primer3) and tested for their mouse specificity using NCBI database. The sequences of the qRT-PCR primer pairs (forward and reverse) used are listed in Supplementary Table S2. The reactions were performed in a 20 µL reaction volume containing 10 pmole of each primer. qRT-PCR was performed with a Fast SYBR Green Master Mix and processed on a 7500 Fast Real-Time PCR System according to manufacturer’s specifications (Applied Biosystems). The following PCR amplification parameters were used: 5 min at 95 °C, followed by 40 cycles of 95 °C for 15 s and 60 °C for 1 min. The dissociation curve was conducted at the end of each PCR reaction. Quantitative data were analysed by average of octuplicates Ct (Cycle threshold) according to the 2^−ΔΔct^ method and normalized versus housekeeping *act*B and *tbp* genes. Data were generated from eight independent experiments and subjected to a an independent one-way ANOVA coupled to Tukey’s multiple-group comparison test (p < 0.05).

### Immunoblotting

Protein extracts (50 μg) from hypoxic and control skeletal muscles, were resolved by SDS-PAGE on 6_14% gradient polyacrylamide gels or 12% homogeneous polyacrylamide gels and transferred onto PVDF membranes. MagicMark XP western protein standard (Thermo Fisher Scientific) was loaded in a separate lane as a molecular weight marker (size range: 20–220 KDa). Blots were incubated with rabbit, goat or mouse polyclonal primary antibodies diluted as follows: anti-HIF-1α (Novus Biologicals) 1:1000, anti-HIF-2α (Novus Biologicals) 1:1000, anti-AMPK (Cell Signaling Technology, CST) 1:1000, anti-pAMPK (CST) 1:1000, anti-PGC1α (Santa Cruz Biotechology, SCBT) 1:500, anti Bnip3 (CST) 1:1000, anti-Becn1 (CST) 1:1000, anti-bcl2 (BD transduction labs) 1:1000, anti-LC3B (CST) 1:1000, anti-Fasn (SCBT) 1:80, anti-Idh1 (SCBT) 1:500, anti-Phd2 and 3 (Novus Biologicals) 1:500 and 1:1000 respectively, anti-Glns (SCBT) 1:500, anti-Gss (SCBT) 1:500, anti-Mdh2 (SCBT) 1:500, anti-Sdha (SCBT) 1:500, anti-Fbp1 (GeneTex) 1:1000, anti-Gna1 (SCBT) 1:500, anti-Uap1 (SCBT) 1:500, anti-Stt3b (Proteintech) 1:1000, anti-Ogt (CST) 1:1000, anti-Oga (Sigma Aldrich) 1:1000. Signals were visualized by chemiluminescence by ECL prime detection kit (GE Healthcare). Images were scanned using ImageQuant LAS 4000 mini digital imaging system (GE Healthcare) and each band was quantitated using ImageQuant Software (GE Healthcare). Data were normalized against the total amount of proteins stained by Sypro Ruby and subjected to an independent one-way ANOVA coupled to Tukey’s multiple-group comparison test. Differences were considered significant at p < 0.05.

### Enzymatic activity assay

Citrate synthase and respiratory chain complexes I, II, and III activities were assessed following thoroughly the method described by M. Spinazzi *et al*.^60^.

## Electronic supplementary material

Supplementary information
