# Supplementary material for: TCA cycle rewiring fosters metabolic adaptation to oxygen restriction in skeletal muscle from rodents and humans
Source: Sci Rep. 2017 Aug 29;7:9723. doi: 10.1038/s41598-017-10097-4 (PMC5575144; doi:10.1038/s41598-017-10097-4)

Supplementary Information

TCA cycle rewiring fosters metabolic adaptation to oxygen restriction in skeletal muscle from rodents and humans.

Daniele Capitanio, Chiara Fania, Enrica Torretta, Agnese Viganò, Manuela Moriggi, Valentina Bravatà, Anna Caretti, Denny Z.H. Levett, Michael P.W. Grocott, Michele Samaja, Paolo Cerretelli, Cecilia Gelfi*

* **Corresponding author**: Professor Cecilia Gelfi, Department of Biomedical Sciences for Health, University of Milan, Via F.lli Cervi 93, 20090, Segrate (MI), Italy. E-mail: cecilia.gelfi@unimi.it Fax: +39(02)21717558

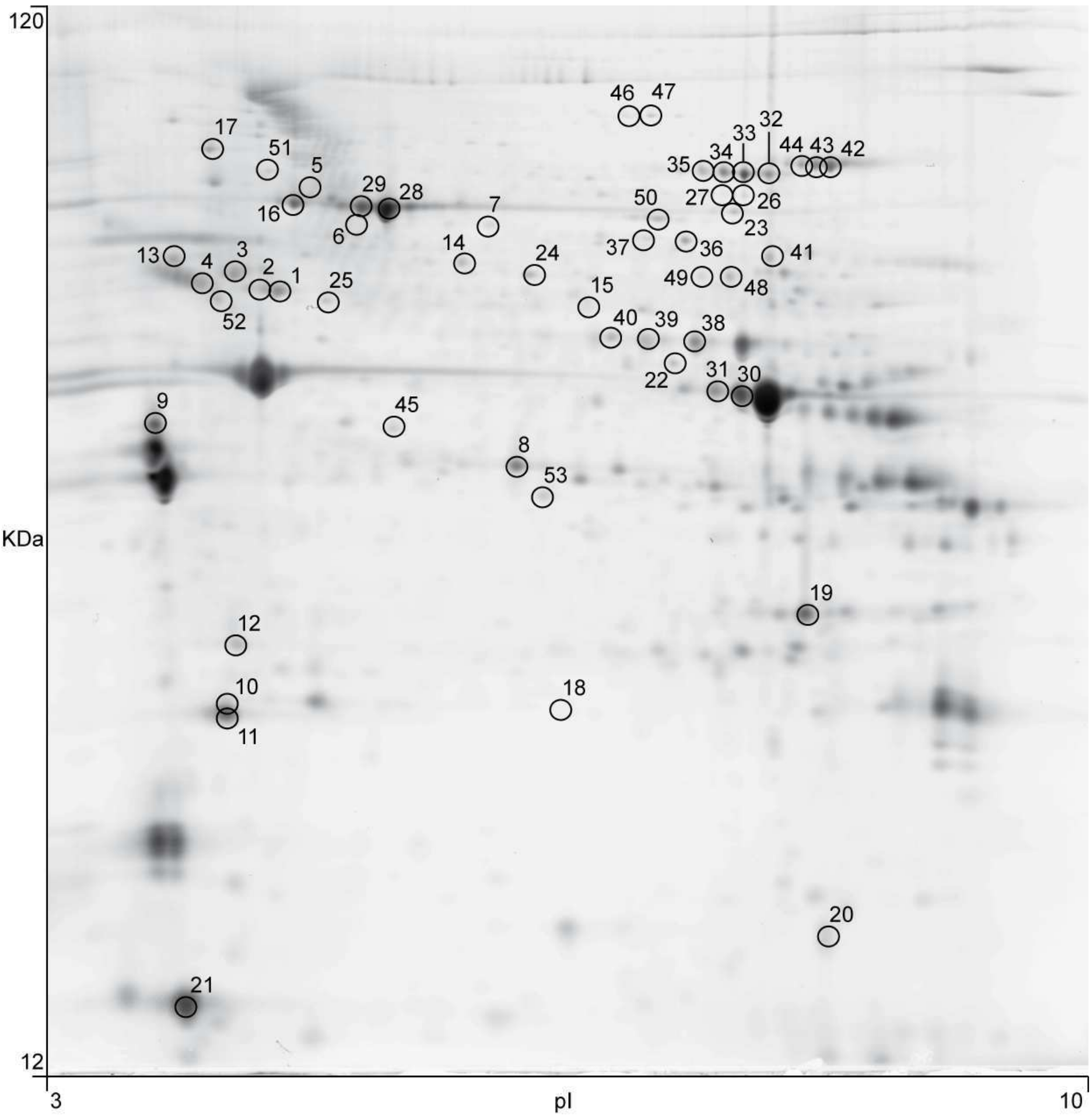

Supplementary Figure S1. Protein profiling by 2-D DIGE. Proteins extracts (50 µg) from each set type were labelled with 400 pmol Cy5 dye (CyDye, GE healthcare), while an internal standard, generated by pooling together an aliquot of all the muscle samples, was labelled with Cy3 dye. Individual samples (40 µg) were combined with an equal amount of internal standard and separated on 24 cm, non linear pH 3-10 gradient IPGstrips (first dimension) and on 20×25 cm2, 12% T, 2,5% C polyacrylamide gels (second dimension). Images were acquired with a 532 nm laser beam and 580 nm emission filter. Protein spots significantly changed (ANOVA + Tukey, p<0.01, n = 5) in normoxic, 2-day and 10-day hypoxia mice extracts compared to their controls and identified by MALDI ToF/ToF mass spectrometry, are numbered in a representative 2-D map and listed in Supplementary Table S1.

Supplementary Table S1. Protein identification by peptide mass fingerprint (PMF). Spots were identified by MALDI/MS. For each identified spot, characterized by a unique spot number assigned during the matching process by the DeCyder software, mass spectrometry parameters (calculated MW and pI, MASCOT score, protein coverage, n° matched over searched peaks) are reported, along with recommended protein name, gene symbol and Uniprot Knowledgebase accession number. To confirm identification, a MS/MS spectrum per protein was collected by MALDI ToF/ToF, as acceptance criterium (in table are reported the sequence of the fragmented peptide and its MS/MS score, mass (m)/charge (z) ratio and charge status (z)).

Function	2D map position (Fig.S1)	UniProtKB accession number	Gene Name	Protein name	Average Ratio 2H/N	% Variation 2H/N	Average Ratio 10H/N	% Variation 10H/N	Theoretical pI	Theoretical MW	MASCOT protein score	Coverage (%)	Matched/ searched peaks	MS/MS	MS/MS score	m/z	z
**Cytoskeletal**	1	P31001	DES	Desmin	-1.74	-43	-2.05	-51	5.21	53366.59	291	51.0	24/33	150VAELYEEEMR159	49.0	1268.5940	1
	2	P31001	DES	Desmin	-2.05	-51	-2.34	-57	5.21	53366.59	297	45.6	25/33	150VAELYEEEMR159	49.2	1268.5940	1
	3	P20152	VIM	Vimentin	/	/	-1.32	-24	5.6	53556.48	330	57.4	25/34	94FANYIDKVR102	34.3	1125.6060	1
	4	P68372	TUBB4B	Tubulin beta-2C chain	/	/	-1.39	-28	4.79	49831.01	171	39.1	17/46	381ISEQFTAMFR390	74.2	1229.6010	1
**Contractile**	5	Q5SX39	MYH4	Myosin-4	-1.28	-22	/	/	5.58	222858.77	215	16.9	35/44	1284LHTESGEFSR1293	65.4	1162.5610	1
	6	Q5SX39	MYH4	Myosin-4	/	/	-1.31	-24	5.58	222858.77	222	16.9	36/45	1284LHTESGEFSR1293	65.8	1162.5614	1
	7	P70402	MYBPH	Myosin-binding protein H	1.51	51	1.36	36	5.66	52588.38	138	35	11/21	230SGDQDSILFIR240	56.4	1250.658	1
	8	Q9QZ47	TNNT3	Troponin T, fast skeletal muscle	1.45	45	/	/	5.26	32109.66	78.5	27.5	7/20	52IPEGEKVDFDDIQK65	91	1632.8184	1
	9	P58774	TPM2	Tropomyosin beta chain	1.45	45	/	/	4.66	32836.70	295	57	22/30	168KLVILEGELER178	93.3	1298.7729	1
	10	P05977	MYL1	Myosin light chain 1/3, skeletal muscle isoform (MLC 1F)	1.28	28	/	/	4.98	20463.32	147	63.3	10/23	81DQGGYEDFVEGLR93	114.7	1483.6579	1
	11	P05977	MYL1	Myosin light chain 1/3, skeletal muscle isoform (MLC 1F)	1.3	30	/	/	4.98	20463.32	91.1	52.7	7/24	81DQGGYEDFVEGLR93	107.5	1483.6579	1
	12	P09542	MYL3	Myosin light chain 3 (MLC 1sb)	/	/	1.59	59	5.03	22290.36	76.7	25.5	5/8	91ALGQNPTQAEVLR103	54	1396.744	1
**Endoplasmic reticulum chaperone**	13	P09103	P4HB	Protein disulfide-isomerase	-1.5	-33	-1.78	-44	4.75	55175.15	250	42.4	19/30	84VDATEESDLAQQYGVR99	152.2	1780.8751	1
	14	P27773	PDIA3	Protein disulfide-isomerase A3	/	/	-1.35	-26	5.69	54267.36	160	51	10/24	86DLGATWVVLGHSER99	54	1539.8002	1
**Cell membrane repair**	15	Q1XH17	TRIM72	Tripartite motif-containing protein 72	/	/	-1.71	-41	6.01	52816.76	157	35.2	16/36	138MQLQEACMR146	28.5	1166.5060	1
**Stress response**	16	Q504P4	HSPA8	Heat shock cognate 71 kDa	/	/	-1.22	-18	5.37	68778.77	250	44.9	23/40	138TVTNAVVTVPAYFNDSQR155	109.5	1982.0060	1
	17	Q71LX8	HSP90AB1	Heat shock protein 84b	/	/	-1.48	-32	4.97	83281.23	273	34	23/27	492EQVANSAFVER502	69.1	1249,635	1
	18	Q99LX0	PARK7	Protein DJ-1	/	/	1.42	42	6.32	20021.31	105	34.9	6/8	146VEKDGLILTSR156	34.4	1230.704	1
**Reversible hydration of carbon dioxide**	19	P16015	CA3	Carbonic anhydrase 3	-1.38	-28	1.95	95	6.97	29235.08	118	53.1	10/36	68VVFDDTYDR76	69.1	1129.5260	1
**Oxygen storage**	20	P04247	MB	Myoglobin	/	/	1.51	51	7.23	16938.49	119	55.2	8/21	18VEADLAGHGQEVLIGLFK35	121.2	1896.0250	1
**Relaxation after contraction**	21	P32848	PVALB	Parvalbumin alpha	1.32	32	1.34	34	5.02	11799.33	110	48.2	7/18	15AIGAFAAADSFDHKK29	125.3	1548.7720	1
**Protein translation**	22	Q8BFR5	TUFM	Elongation factor Tu, mitochondrial	/	/	-1.25	-20	6.2	44970.87	187	45.4	15/27	105GITINAAHVEYSTAAR120	106.5	1673.8640	1
**Mitochondrial structural protein**	23	Q2YDW0	IMMT	Immt protein	/	/	-1.28	-22	5.72	50155.67	113	39.5	12/38	36VVSQYHELVVQAR48	61.4	1527.8370	1
**Coagulation**	24	Q8K0E8	FGB	Fibrinogen beta chain	-1.72	-42	-1.44	-31	6.4	52498.91	224	51.6	22/51	264TENGGWTVIQNR275	86.9	1374.6720	1
	25	Q8VCM7	FGG	Fibrinogen gamma chain	-1.89	-47	-1.56	-36	5.55	46671,09	130	40.8	12/33	188ESGLYFIRPLK198	43.9	1322.7520	1
**Transport**	26	Q921I1	TF	Serotransferrin	-1.75	-43	-1.39	-28	6.81	74881.46	234	34.9	22/37	332LYLGHNYVTAIR343	58.8	1419.7980	1
	27	Q921I1	TF	Serotransferrin	-1.44	-31	/	/	6.81	74881.46	192	30.8	20/42	332LYLGHNYVTAIR343	80.8	1419.7960	1
	28	P07724	ALB	Serum albumin	-1.34	-25	-1.48	-32	5.75	68692.51	385	53.1	30/38	439APQVSTPTLVEAAR452	80.8	1439.8130	1
	29	P07724	ALB	Serum albumin	/	/	-1.2	-17	5.75	68692.51	414	59.7	34/48	439APQVSTPTLVEAAR452	112.5	1438.7880	1
**Energy transfer**	30	P07310	CKM	Creatine kinase M-type	-1.65	-39	/	/	6.58	43044.97	281	48.8	19/23	139GYTLPPHCSR148	55.4	1187.5730	1
	31	P07310	CKM	Creatine kinase M-type	-1.44	-31	/	/	6.58	43044.97	207	49.6	18/36	224SFLVWVNEEDHLR236	97.7	1642.8104	1
**Glycogen metabolism**	32	Q9WUB3	PYGM	Glycogen phosphorylase, muscle form	-1.59	-37	-1.52	-34	6.65	97155.11	370	42.4	33/40	51DYYFALAHTVR61	76.6	1355.6899	1
	33	Q9WUB3	PYGM	Glycogen phosphorylase, muscle form	-1.74	-43	-1.58	-37	6.65	97155.11	262	34.6	28/44	51DYYFALAHTVR61	90.4	1355.6940	1
	34	Q9WUB3	PYGM	Glycogen phosphorylase, muscle form	-1.52	-34	-1.36	-26	6.65	97155.11	362	43.1	32/38	51DYYFALAHTVR61	76.8	1355.6740	1
	35	Q9WUB3	PYGM	Glycogen phosphorylase, muscle form	-1.32	-24	/	/	6.65	97155.11	311	40.7	31/44	51DYYFALAHTVR61	96	1355.7030	1
**Glycolytic enzyme**	36	Q5RJV4	PGM2	Phosphoglucomutase 2	1.32	32	/	/	6.14	61384.18	218	45.9	17/27	278SGEHDFGAAFDGDGDR293	123	1652.6870	1
	37	Q5RJV4	PGM2	Phosphoglucomutase 2	-1.51	-34	-1.5	-33	6.14	61384.18	128	32.2	11/23	278SGEHDFGAAFDGDGDR293	143.4	1652.6600	1
	38	Q4FK59	ENO3	Enolase	-1.57	-36	-1.5	-33	6.29	46997.77	80.3	23	8/24	359LAQSNGWGVMVSHR372	86.7	1541.8101	1
	39	Q4FK59	ENO3	Enolase	-1.39	-28	-1.3	-23	6.29	46997.77	201	40.3	13/16	359LAQSNGWGVMVSHR372	87.7	1541.7900	1
	40	Q4FK59	ENO3	Enolase	-1.37	-27	/	/	6.29	46997.77	191	66.2	13/17	359LAQSNGWGVMVSHR372	83	1541.7841	1
	41	P52480	PKM	Pyruvate kinase isozymes M1/M2	-1.42	-30	-1.44	-31	7.42	57713.70	147	33.5	14/29	476DAVLNAWAEDVDLR489	87.3	1586.7760	1
**TCA cycle enzyme/OXPHOS**	42	Q99KI0	ACO2	Aconitate hydratase, mitochondrial	-1.33	-25	-1.43	-30	7.4	82463.88	165	26.3	19/43	634NAVTQEFGPVPDTAR648	129.8	1601.8380	1
	43	Q99KI0	ACO2	Aconitate hydratase, mitochondrial	/	/	-1.37	-27	7.4	82463.88	246	39.4	24/43	634NAVTQEFGPVPDTAR648	110	1601.8260	1
	44	Q99KI0	ACO2	Aconitate hydratase, mitochondrial	/	/	-1.27	-21	7.4	82463.88	273	41.5	26/44	634NAVTQEFGPVPDTAR648	134.5	1601.7820	1
	45	Q9D6R2	IDH3A	Isocitrate dehydrogenase [NAD] subunit alpha	/	/	-1.57	-36	5.6	36707.29	108	25.7	10/25	179IAEFAFEYAR188	77.5	1216.6130	1
	46	Q60597	OGDH	2-oxoglutarate dehydrogenase, mitochondrial	/	/	-1.26	-21	6.05	111839.51	161	19.3	17/28	958NQGYYDYVKPR968	49.9	1402.7020	1
	47	Q60597	OGDH	2-oxoglutarate dehydrogenase, mitochondrial	/	/	-1.27	-21	6.05	111839.51	143	13.3	13/16	958NQGYYDYVKPR968	65.4	1402.6949	1
	48	O08749	DLD	Dihydrolipoyl dehydrogenase, mitochondrial	/	/	-1.38	-28	6.43	50242.71	141	21.8	10/13	483VCHAHPTLSEAFR495	73.6	1524.7380	1
	49	O08749	DLD	Dihydrolipoyl dehydrogenase, mitochondrial	/	/	-1.38	-28	6.43	50242.71	155	21.8	11/14	483VCHAHPTLSEAFR495	74	1524.7377	1
	50	Q8K2B3	SDHA	Succinate dehydrogenase [ubiquinone] flavoprotein subunit, mitochondrial	1.17	17	-1.22	-18	6.32	68032.07	256	43.4	20/31	148FISDKDASVVGFFR161	95.1	1587.8290	1
	51	Q91VD9	NDUFA1	NADH dehydrogenase (ubiquinone) Fe-S protein 1	1.13	13	-1.19	-16	5.24	77182.59	282	36.7	24/30	409FEAPLFNAR417	72.6	1064.5640	1
	52	P56480	ATP5B	ATP synthase subunit beta, mitochondrial	1.15	15	/	/	4.99	51749.20	295	51	21/26	226AHGGYSVFAGVGER239	116.3	1406.6689	1
	53	P14152	MDH1	Malate dehydrogenase, cytoplasmic	1.19	19	/	/	6.16	36379.97	99.3	35.3	9/24	299FVEGLPINDFSR310	86.7	1393.7061	1

Supplementary Table S2. Primer sequences used for qRT-PCR analyses.

Gene symbol	Gene ID	Full name	Forward primer 5'>3'	Reverse primer 5'>3'	Template size (base pairs)
AMPKα1	105787	Protein kinase, AMP-activated, alpha 1	agatcggccactacatcctg	atttttcccaccacgtcaag	148
BAX	12028	BCL2-associated X protein	gaaccatcatgggctgga	cagcccatcttcttccagat	178
BCL2	12043	B cell leukemia/lymphoma 2	gccgagaagaagggagaatc	tgctttgcattcttggatga	105
BECN1	56208	Beclin 1, autophagy related	agcctctgaaactggacacg	aaatggctcctctcctgagtt	140
BNIP3	12176	BCL2/adenovirus E1B interacting protein 3	tttgtttcactgtcccacactt	ttgctccaaatgaaagggata	120
BNIP3L	12177	BCL2/adenovirus E1B interacting protein 3-like	tgagaagcaggcttcgtttt	gctgcttcaccgtgagagtt	125
Casp3	12367	Caspase 3	gggggcgtgtttctgttttgt	ctgcattgctaggcagtggt	116
EPAS1	13819	Endothelial PAS domain protein 1 (HIF2α)	ctaagtggcctgtgggtgat	gtgtcttggaaggcttgctc	232
HIF1α	15251	Hypoxia inducible factor 1, alpha subunit	tggagatgctggctccctat	atcagtggtggcagttgtgg	185
LC3A	66734	Microtubule-associated protein 1 light chain 3 alpha	cggcttctgagtcaagagga	ggacctacccaggatgtggt	182
LC3B	67443	Microtubule-associated protein 1 light chain 3 beta	ccagaaactgagctccatgc	cccctgacactgctcttctg	187
LKB1	20869	Serine/threonine kinase 11	ctactccgagggatgttgga	gataggtacgagcgcctcag	114
mTOR	56717	Mechanistic target of rapamycin (serine/threonine kinase)	tccaggaggacatttgttca	cagcatatccctccctcact	113

Supplementary Data. Full-lenght blot images (N, normoxic; 2H, 2-day hypoxia; 10H, 10-day hypoxia; SL, sea level; EE, Everest expedition; MR, Mt. Rosa). Red rectangles highlight bands that are shown in manuscript figures.

A. HIF-1α and HIF-2α signalling

HIF-1alpha (120 KDa)

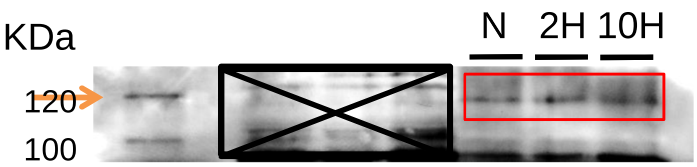

HIF-2alpha (96 KDa)

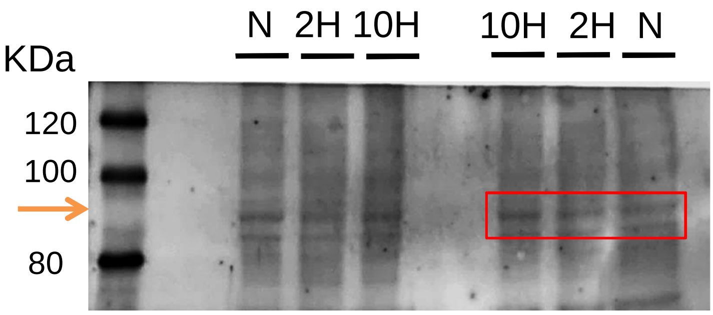

B. Energy sensing and energetic metabolism regulators

pAMPK (62 KDa)

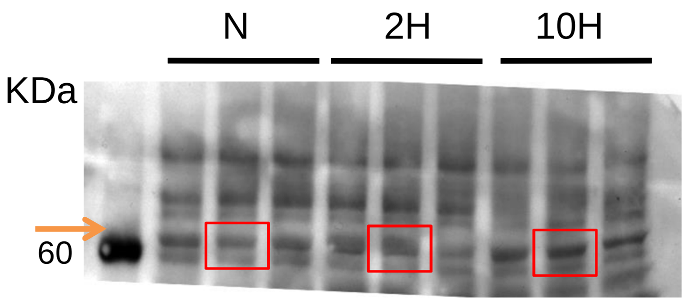

AMPK (62 KDa)

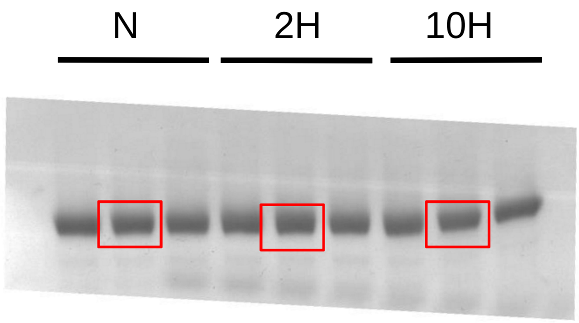

PGC1alpha (90 KDa)

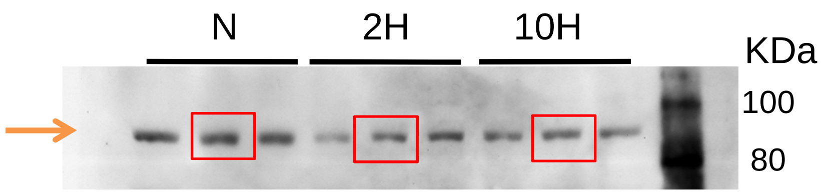

C. Citrate utilization

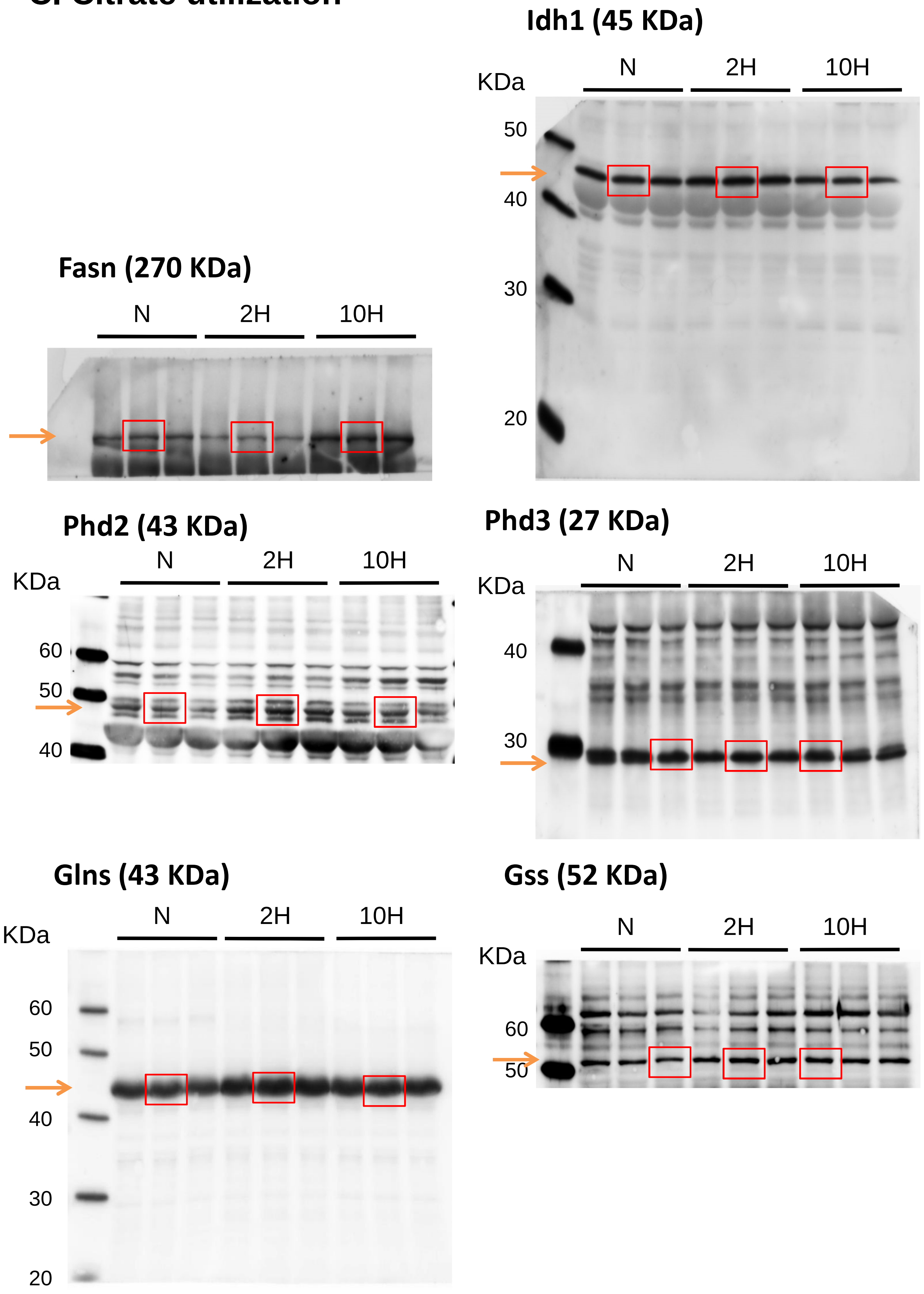

Sdha (68 KDa)

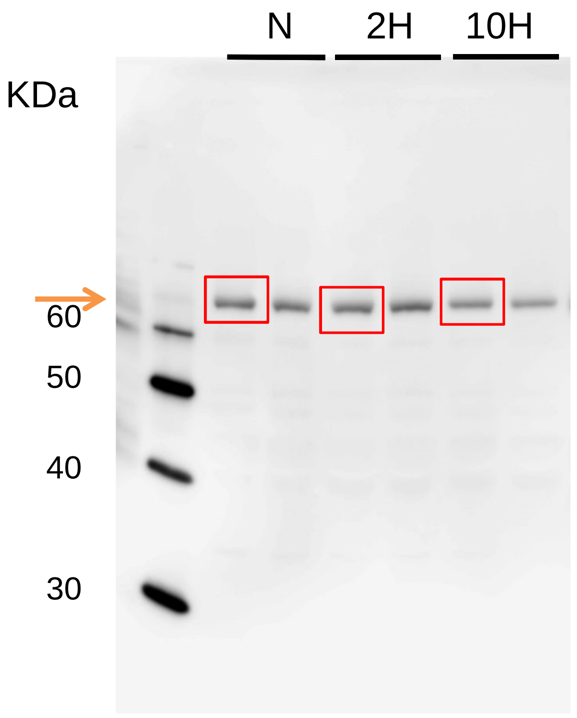

Mdh2 (36 KDa)

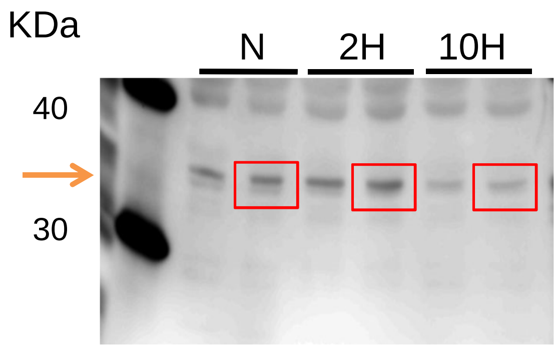

D. Hexosamine biosynthetic pathway

Fbp1 (37 KDa)

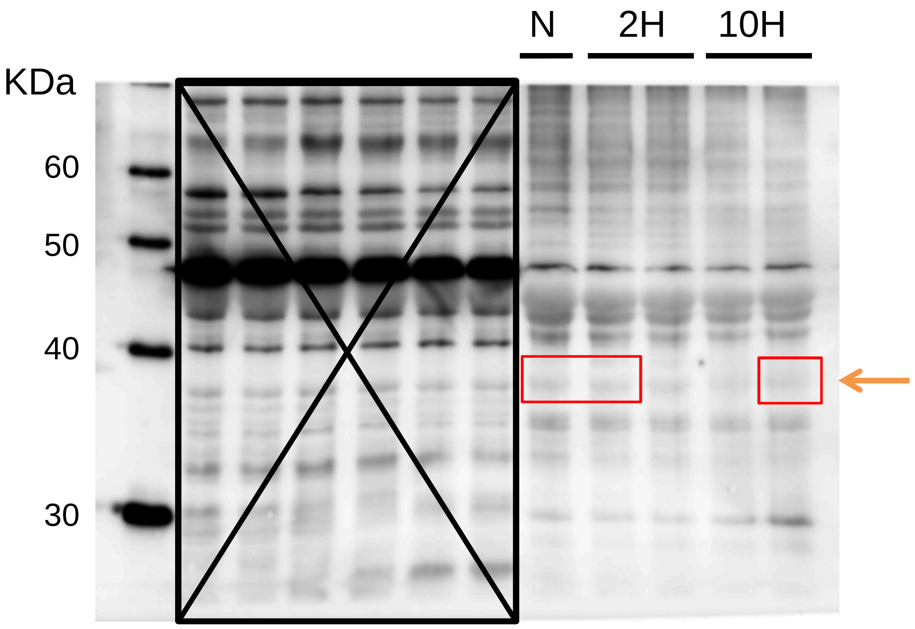

Uap1 (56-58 KDa)

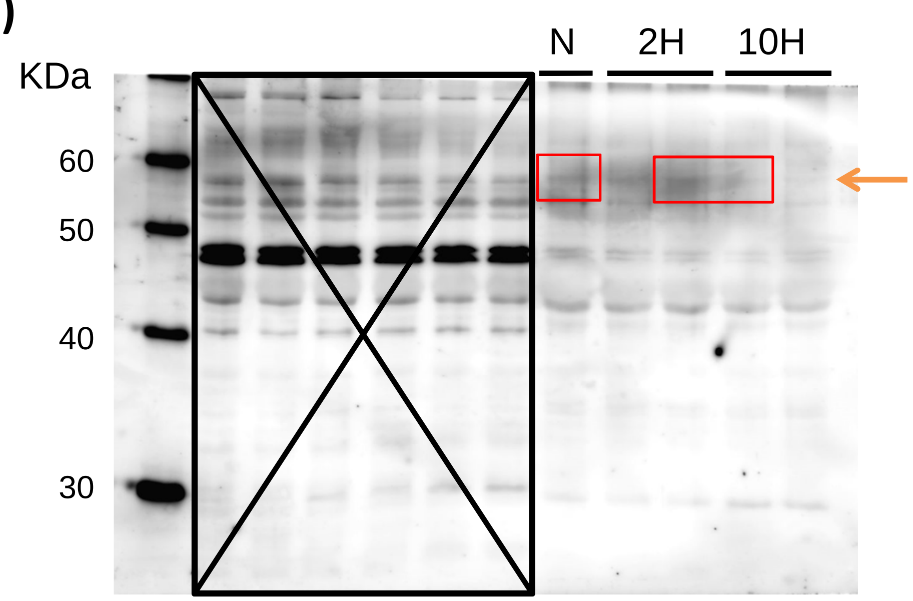

Gna1 (23 KDa)

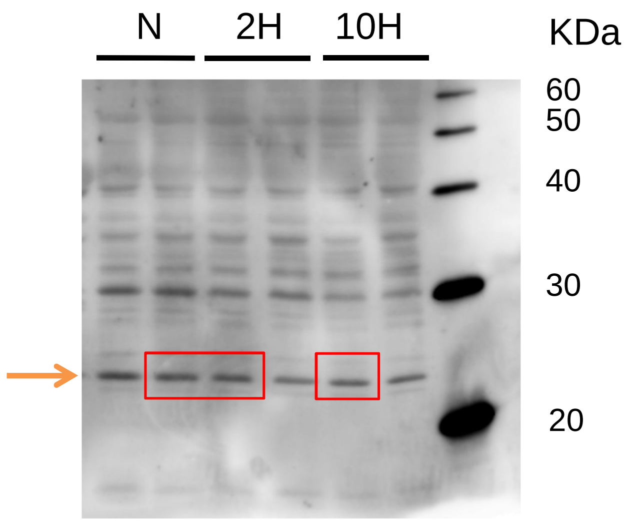

Ogt (110 KDa)

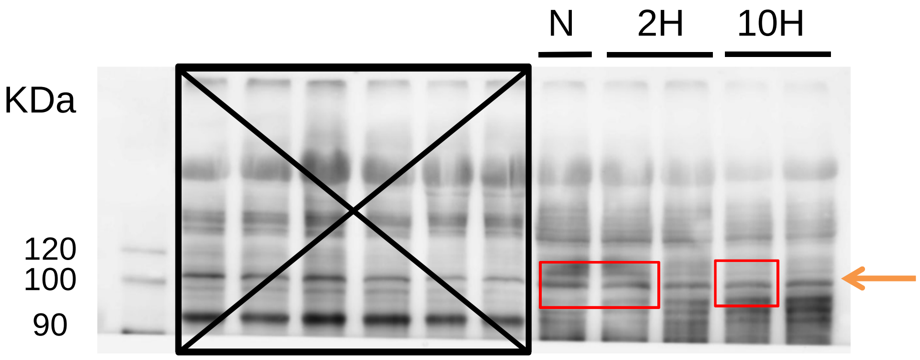

Oga (130 KDa)

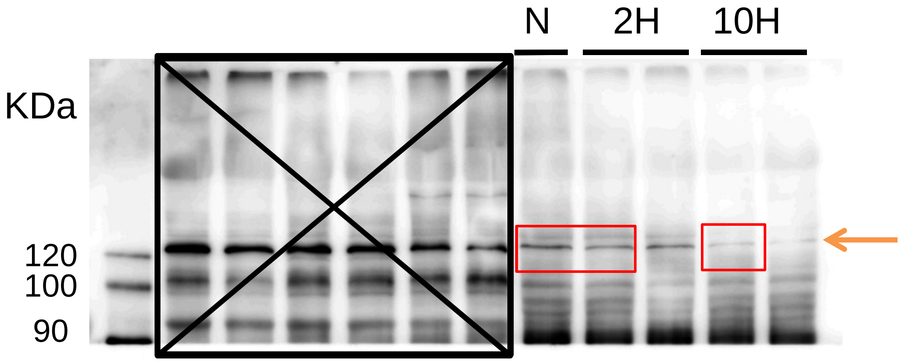

Stt3b (94-97 KDa)

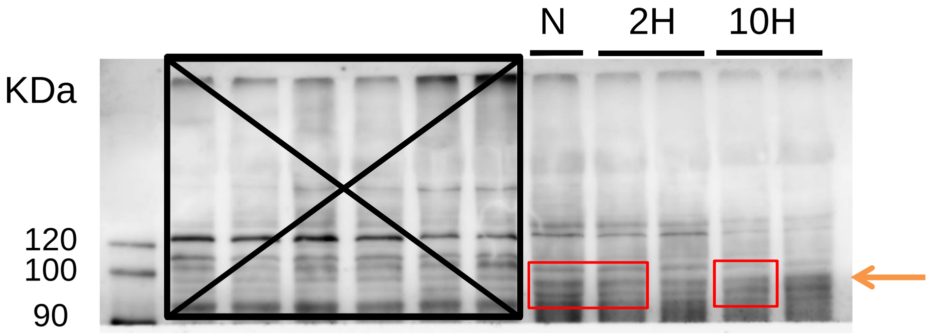

E. Autophagy

Bnip3 (22-30 KDa)

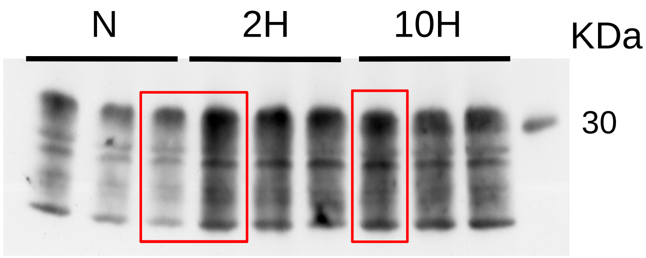

Bcl2 (26 KDa)

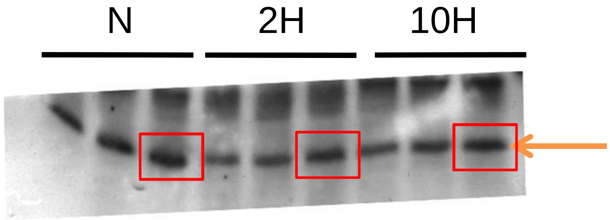

Becn1 (60 KDa)

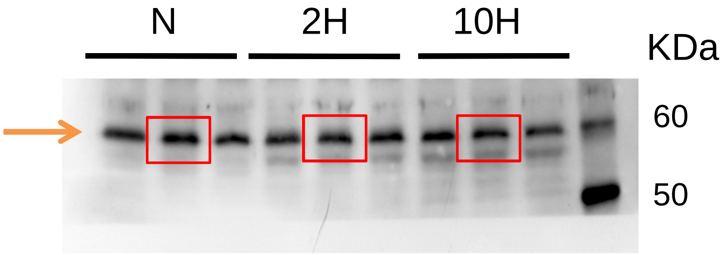

LC3B-I/LC3B-II (16/14 KDa)

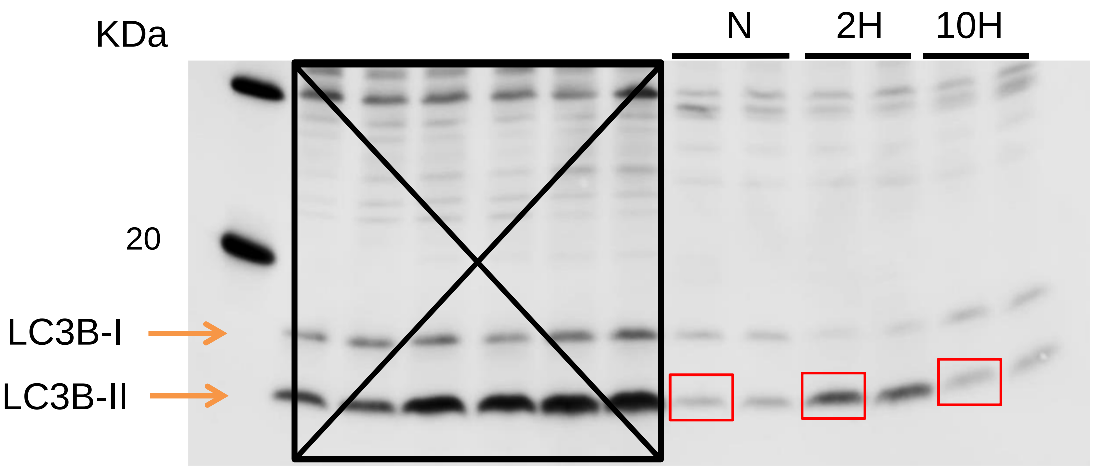

F. TCA cycle rewiring in hypoxic human skeletal muscle

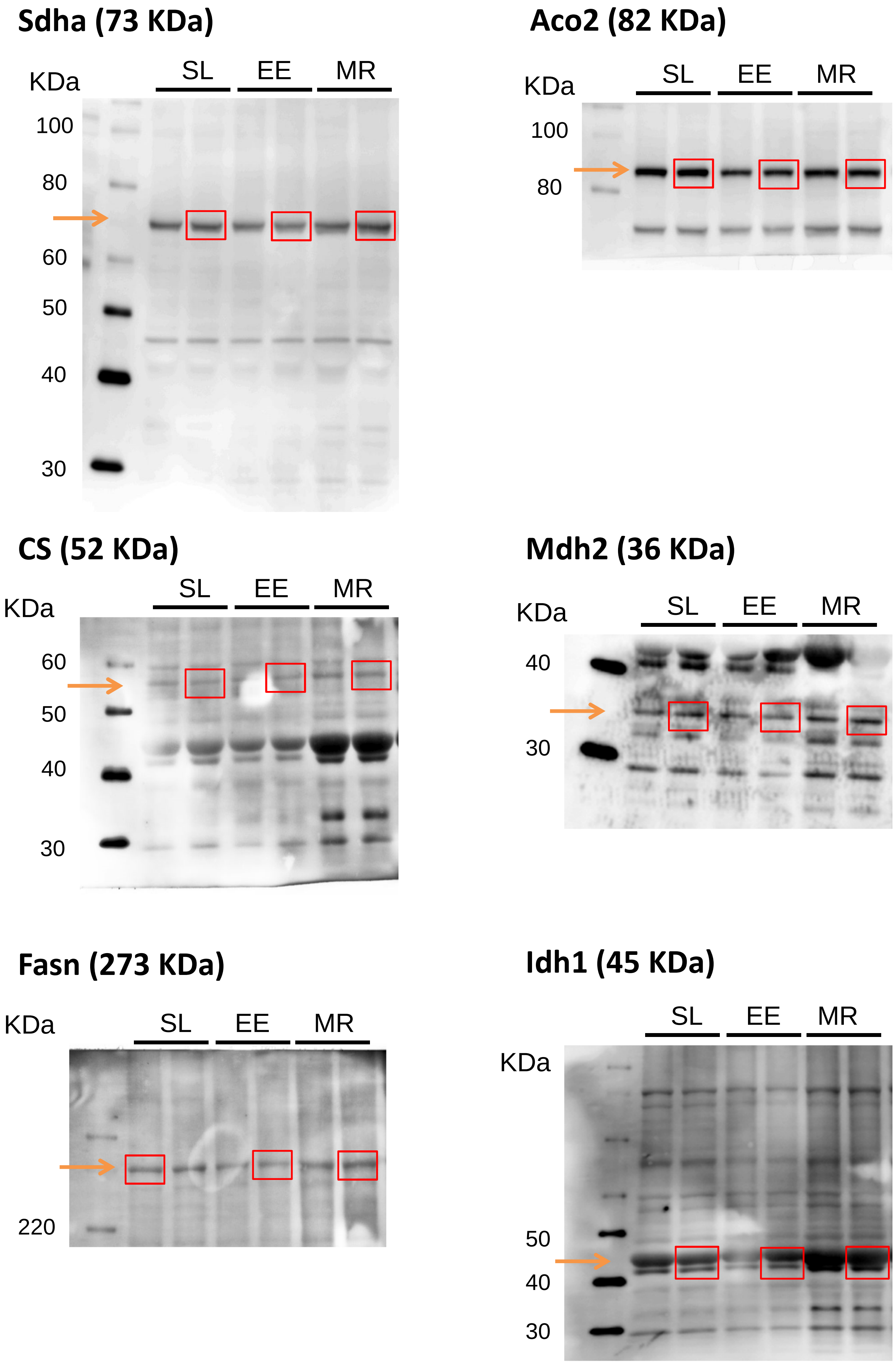

Supplement: Supplementary file 1 — Supplementary information [file 41598_2017_10097_MOESM1_ESM.pdf]
